# Revision of the genus *Dercetina* from Taiwan and their similar species, with description of a new species from Myanmar (Insecta, Chrysomelidae, Galerucinae)

**DOI:** 10.3897/zookeys.323.5195

**Published:** 2013-08-13

**Authors:** Chi-Feng Lee, Jan Bezděk

**Affiliations:** 1 Applied Zoology Division, Taiwan Agricultural Research Institute, 189 Chung-Cheng Road, Wufeng, Taichung 413, Taiwan; 2 Mendel University, Department of Zoology, Zemědělská 1, 613 00 Brno, Czech Republic

**Keywords:** *Dercetina*, *Arthrotus*, Taiwan, taxonomic revision

## Abstract

Species of the genus *Dercetina* Gressitt & Kimoto, 1963 in Taiwan are revised. *Dercetina azumai* Gressitt and Kimoto, 1966, *Dercetina itoi* Kimoto, 1969, and *Dercetina shirozui* Kimoto, 1969 are redescribed. *Dercetina chinensis* (Weise, 1889), *Dercetina taiwana* (Chûjô, 1938), and *Dercetina unifasciata* (Allard, 1889) are removed from synonymy with *Dercetina flavocincta* (Hope, 1831). *Dercetina flavocincta* and *Dercetina nakanei* Kimoto, 1969 are transferred to the genus *Arthrotus* Motschulsky, 1858. Lectotypes are designated for *Dercetis taiwana* Chûjô, 1938, and *Antipha varipennis* Jacoby, 1890. The synonymies of *Antipha flavofasciata* Baly, 1879 and *Dercetes femoralis* Weise, 1922 with *Arthrotus flavocincta* (Hope, 1831), *Antipha varipennis* Jacoby, 1890 with *Dercetina chinensis* (Weise, 1889) are supported. A new species, *Dercetina barclayi* **sp. n.** which was confused with *Dercetina flavocincta*, is described from Myanmar.

## Introduction

*Dercetina* Gressitt & Kimoto, 1963 is very similar to the genus *Arthrotus* Motschulsky, 1858. These genera differ from each other only by the structure of male antenna: the antennomere III is about twice longer than antennomere II in *Dercetina*, while antennomeres II and III are subequal in length in *Arthrotus*. Thus females are impossible to assign to either genus if they are not associated with males. Moreover, most members of *Dercetina* have similar shapes of male aedeagi and some species have great color variation. These factors have caused taxonomic confusion in *Dercetina* and *Arthrotus*. To solve this problem, examination of extensive collections and evaluation of characters other than the external morphology of the male aedeagi are necessary. The examination of endophallic sclerites, which we studied here for the first time in *Dercetina* and *Arthrotus*, seems to be very helpful for resolving many taxonomical problems in both genera.

*Dercetina* is an Asian genus and comprises 88 species (Wilcox 1971, 1975, Bezděk, unpublished data), of which 23 species are distributed in the Palearctic region (Beenen 2010). Five species were recorded from Taiwan (Kimoto and Takizawa 1997). *Dercetes flaviventris* (Jacoby, 1890) was the first species to be recorded from Taiwan by Weise (1922). Later Chûjô (1938) described the new species *Dercetis taiwana*. Kimoto (1969) indicated that the record of *Dercetes flaviventris* was a misidentification and described a new species as *Dercetina itoi*. In addition to *Dercetina itoi*, two more new species were described (*Dercetina shirozui* Kimoto, 1969 and *Dercetina nakanei* Kimoto, 1969) and one new country record for Taiwan (*Dercetina azumai* Kimoto & Gressitt, 1966).

*Dercetina taiwana* (Chûjô, 1938) has a complicated nomenclatural history. It was synonymized with *Dercetina chinensis* (Weise, 1889) by Gressitt and Kimoto (1963). *Antipha varipennis* Jacoby, 1890 was also regarded as a junior synonym in the same paper. Later, *Dercetina chinensis* was synonymized with *Dercetina flavocincta* (Hope, 1831) by Kimoto (1989b). *Antipha unifasciata* Allard, 1889 and *Dercetes femoralis* Weise, 1922 were also regarded as junior synonyms of *Dercetina flavocincta* in the same paper. *Antipha flavofasciata* Baly, 1879 was synonymized with *Dercetina flavocincta* by Maulik (1936). To clarify the status of all available names, the types of them were re-examined and diagnostic characters examined.

## Material and methods

To study specimens and prepare drawings of the adult reproductive systems, the abdomens of adults were separated and boiled in a 10% KOH solution, cleared in distilled water, and then mounted on slides with glycerin. Slides were examined using a Leica M165 stereomicroscope, and figures were drawn using a Nikon ECLIPSE 50i microscope.

Studied specimens have been deposited at the following institutes and museums and

BMNH The Natural History Museum, London, UK [Maxwell V. L. Barclay]

BPBM Bernice P. Bishop Museum, Honolulu, USA [Shepherd Myers]

ISNB Institut royal des Sciences Naturelles de Belgique, Bruxelles, Belgium [Pol Limbourg]

KMNH Kitakyushu Museum of Natural History and Human History, Kitakyushu, Japan [Kyoichiro Ueda]

KUEC Faculty of Agriculture, Kyushu University, Fukuoka, Japan [Osamu Tadauchi]

MCZC Museum of Comparative Zoology, Harvard University, Massachusetts, USA [Philip D. Perkins]

MNHUB Museum für Naturkunde, Leibniz-Institut für Evolutions- und Biodiversitätsforschung an der Humboldt-Universität zu Berlin, Berlin, Germany [Joachim Willer, Johannes Frisch]

NHRS Naturhistoriska Riksmusset, Stockholm, Sweden [Johannes Bergsten]

SDEI Senckenberg Deutsches Entomologisches Institut, Müncheberg, Germany [Stephan Blank]

TARI Taiwan Agricultural Research Institute, Taichung, Taiwan.

USNM National Museum of Natural History, Smithsonian Institution, Washington DC, USA [Alexander Konstantinov]

## Results

### Dercetina azumai

Kimoto & Gressitt, 1966

http://species-id.net/wiki/Dercetina_azumai

Dercetina azumai Kimoto & Gressitt, 1966: 534 (Japan: Iriomote island); Kimoto 1969: 66 (Taiwan); Kimoto 1989a; 260 (Taiwan).

#### Type series.

*Dercetina azumai*: Holotype ♀ (KUEC): “RYUKYU IS. Iriomote I. Ushiku-mori 11.III.1964 / S. Kimoto Collector / Japan-U. S. Co-op. Sci. Programme (yellow label) / HOLOTYPE DERCETINA AZUMAI J. L. GRESSITT (red label)”.

#### Material examined.

**TAIWAN:** 1♂, Kaoshiung, Chuyunshan trail, 1.III.2009, leg. U. Ong (TARI); 1♀, Kaoshiung, Taoyuan, 3.VII.2009, leg. S.-F. Yu (TARI); 5♂♂, 12♀♀, Kaoshiung, Tengchih (= Shihshan trail), 2-5.VI.2008, leg. C.-F. Lee (TARI); 3♂♂, 16♀♀, same locality, 2.X.2008, leg. M.-H. Tsou (TARI); 6♀♀, same locality, 1–3.X.2008, leg. M.-H. Tsou (TARI); 1♂, 1♀, same locality, 5.II.2009, leg. M.-H. Tsou (TARI); 1♂, 1♀, same locality, 26.V.2009, leg. C.-F. Lee (TARI); 1♂, same locality, 4.VII.2011, leg. M.-H. Tsou (TARI); 1♀, Pingtung, Jinshuiying, 12.IV.2012, leg. C.-F. Lee (TARI); 2♀♀, Pingtung, Tahanshan, 22.I.2009, leg. S.-F. Yu (TARI); 1♀, same locality, 24.I.2009, leg. M.-H. Tsou (TARI); 1♀, same locality, 8.V.2009, leg. U. Ong (TARI); 1♂, same locality, 21.VII.2009, leg. J.-C. Chen (TARI); 6♂♂, 14.VIII.2011, leg. Y.-T. Wang (TARI); 1♀, same locality, 6.VI.2012, leg. C.-F. Lee (TARI); 1♂, same locality, 19.VII.2012, leg. C.-F. Lee (TARI); 2♂♂, Taitung, Motien, 23.VI.2010, leg. S.-F. Yu (TARI); 1♂, same locality, 19.VI.2011, leg. C.-F. Lee (TARI).

#### Diagnosis.

*Dercetina azumai* is similar to *Dercetina shirozui* with metallic green elytra but differs by the yellowish brown head, prothorax, meso- and metathoracic ventrites (in contrast with metallic green head, prothorax, meso- and metathoracic ventrites in *Dercetina shirozui*).

#### Redescription.

**Figures 1–4.**
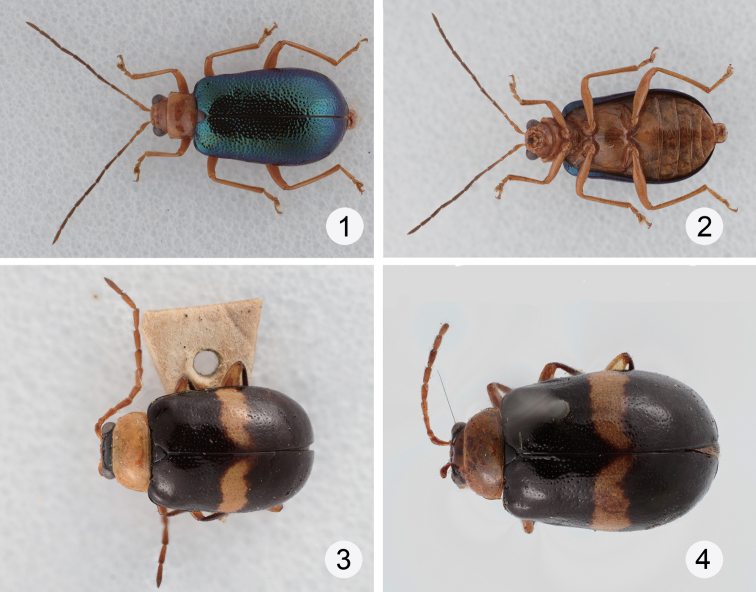
Habitus of *Dercetina* species. **1** *Dercetina azumai*, dorsal view **2** *Dercetina azumai*, ventral view **3** *Dercetina barclayi* sp. n., dorsal view **4** *Dercetina barclayi* sp. n., ventral view.

Color (Figs 1–2) yellowish brown except eye black; antennomeres III-XI dark brown; elytron metallic green or blue. Head shagreened and impunctate. Pronotum transverse, 1.4–1.5 times wider than long, disc with a pair of deep fovea, and scattered prominent and fine punctures; lateral margin sinuate, narrowed posterior, anterior margin slightly concave, posterior margin slightly rounded. Elytra more or less widened posterior, apex convergent rounded, 1.6–1.7 times longer than wide, disc with densely prominent punctures; epipleurae with scattered prominent prunctures.

**Figures 5–11.**
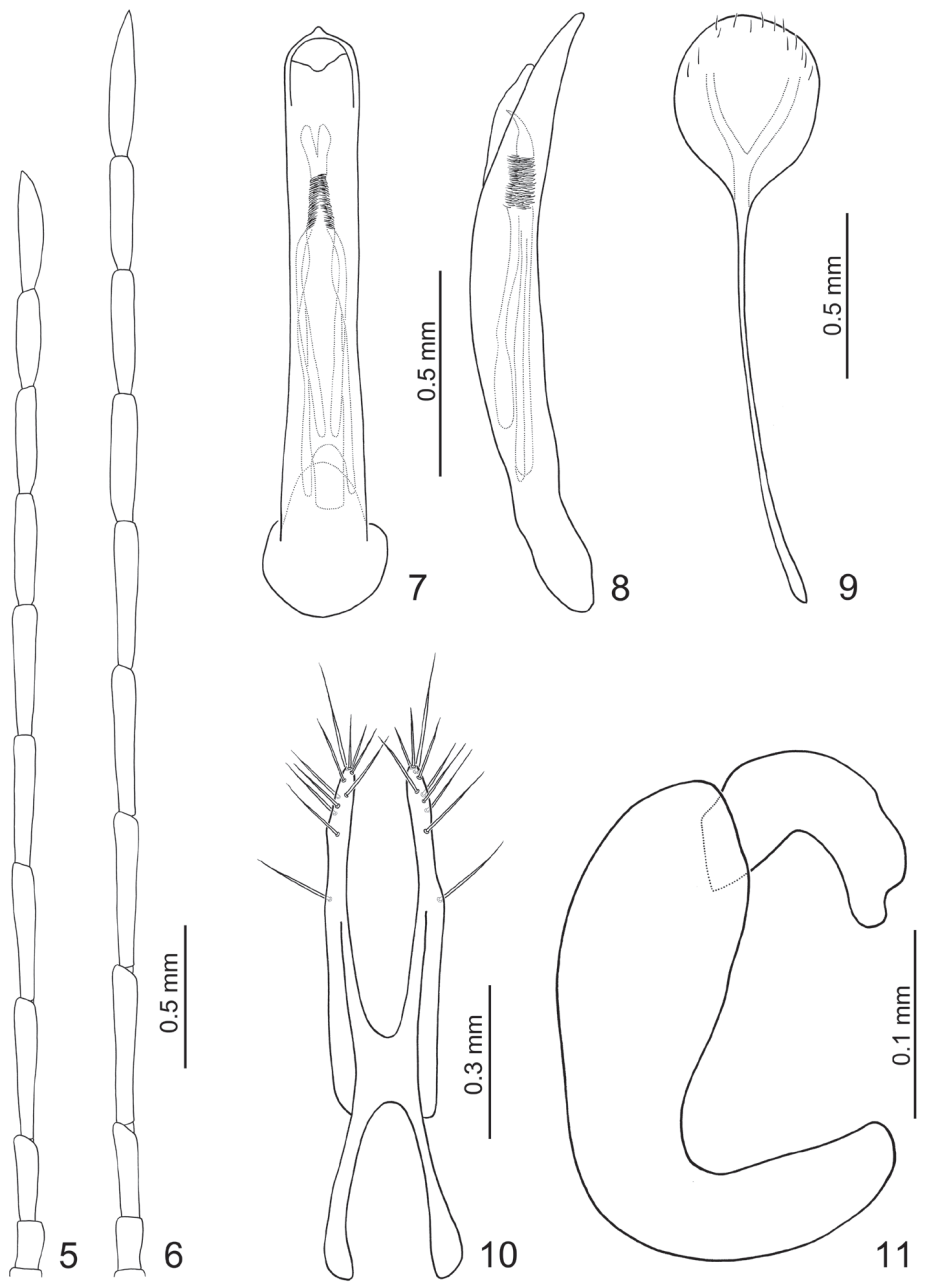
*Dercetina azumai*. **5** Antenna, male **6** Antenna, female **7** Aedeagus, dorsal view **8** Aedeagus, lateral view **9** Sternite VIII **10** Gonocoxae **11** Spermatheca.

**Male.** Length 4.9–5.6 mm, width 2.1–2.4 mm. Antenna filiform (Fig. 5), ratio of length of antennomeres III to XI about 1.0: 1.6: 1.6: 1.6: 1.6: 1.3: 1.2: 1.1: 1.4; ratio of length to width of antennomeres III to XI about 3.9: 6.4: 6.1: 6.0: 6.2: 5.1: 4.8: 4.1: 5.1. Penis (Fig. 7) extremely slender, about 8.9 times longer than wide, parallel-sided, basally widened, apex pointed; tectum membranous, with scattered stout membranous setae; weakly curved in lateral view (Fig. 8); endophallic sclerite elongate, about 0.7 times as long as penis, apex curved in lateral view, bifurcate, with a cluster of dense setae near apex; a pair of hooked dorsal slclerite connected at apical 1/5, an elongate sclerite between dorsal sclerite and ventral sclerite, connected at apical 1/3; ventral sclerite with base deeply bifurcate.

**Female.** Length 5.5–6.8 mm, width 2.6–3.3 mm. Ratio of length of antennomeres III to XI about 1.0: 1.7: 1.6: 1.6: 1.5: 1.4: 1.3: 1.3: 1.5; ratio of length to width of antennomeres III to XI about 3.8: 6.3: 6.1: 5.9: 5.6: 5.1: 4.9: 4.9: 5.7 (Fig. 6). Sternite VIII (Fig. 9) weakly sclerotized laterally and apically, with scattered setae along lateral and apical margin, spiculum extremely slender. Spermathecal receptaculum (Fig. 11) weakly swollen; pump narrow and moderately curved, apex broadly rounded; spermathecal duct long, strongly curved, deeply projecting into receptaculum. Gonocoxae (Fig. 10) widely connected at middle, about 4.4 times longer than wide, curved inwards at apical 1/3, with one long seta at apical 1/3, eleven setae at apex.

#### Host plants.

Myrsinaceae: *Embelia lenticellata* Hayata; Saxifragaceae: *Hydrangea angustipetala* Hayata.

#### Distribution.

**Figures 12–15.**
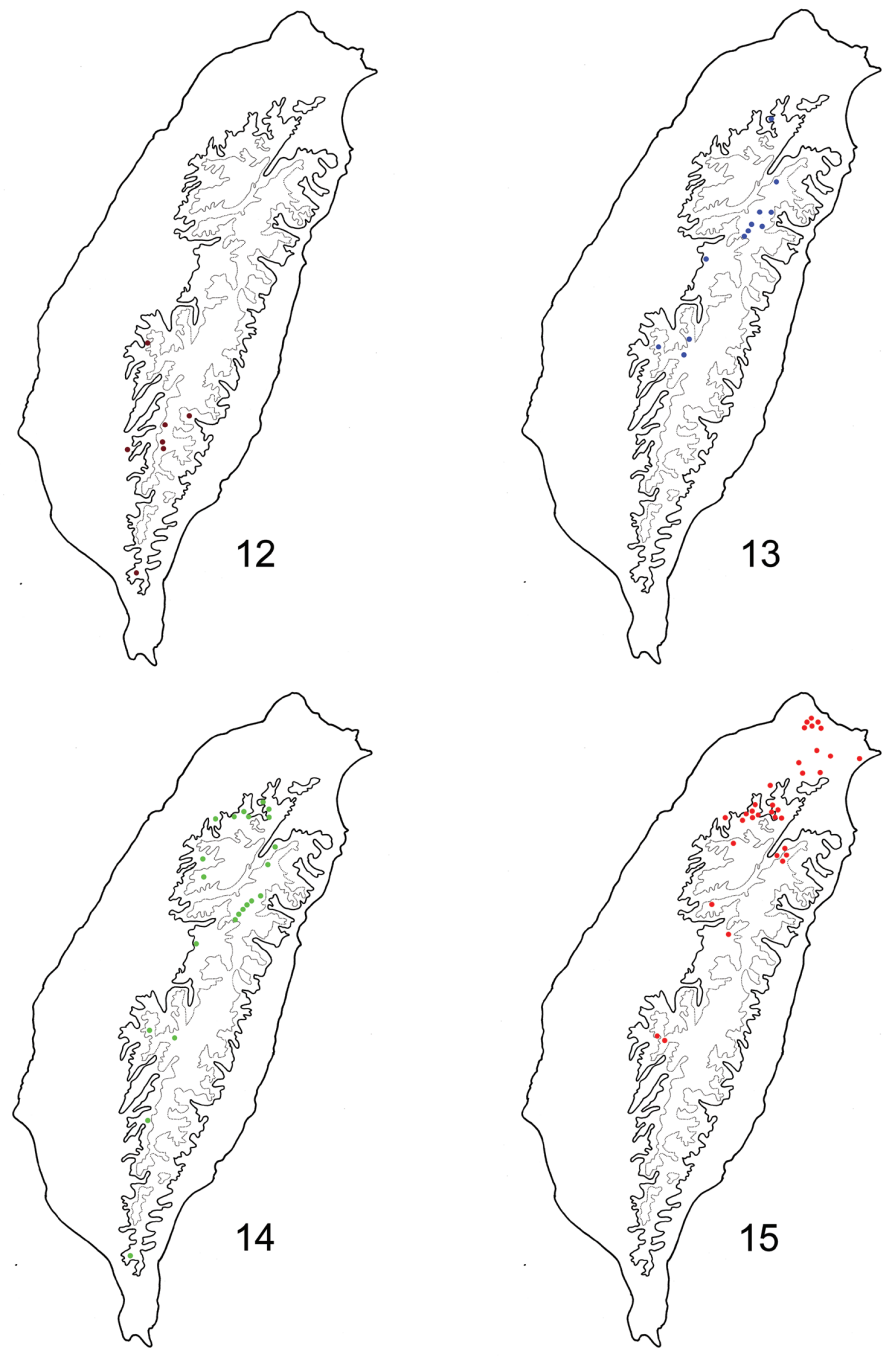
Distribution map of *Dercetina* species, solid line: 1000 m, broken line: 2000 m. **12** *Dercetina azumai* **13** *Dercetina itoi* **14** *Dercetina shirozui* **15** *Dercetina taiwana*.

Japan (Iriomote island) and Taiwan. This species occurs in mountains (1000–2000 m) of southern Taiwan (Fig. 12) but locally abundant.

### Dercetina barclayi sp. n.

http://zoobank.org/AD8891A6-94FF-483F-B6D8-70378076A04E

http://species-id.net/wiki/Dercetina_barclayi

#### Type series.

Holotype ♂ (BMNH): “Doherty / Birmah RubyMes / Fry Coll. 1905. 100.”. Paratypes: 1♂, same as holotype (BMNH); 1♂, 2♀♀: “Ruby Mines. Burma. 5.500 to 7.500 ft. 1904-150.” (BMNH); 4♂♂, 1♀: “Ruby Mines U. M. / Gift of F. G. Bowditch” (USNM).

#### Diagnosis.

This new species is similar to *Dercetina taiwana* but differs by yellowish brown antennae, no color variation, wider penis, and with longer endophallic sclerites.

#### Description.

Color (Figs 3–4) bluish black, prothorax, coxae, and tibiae yellowish brown; elytron with one trasverse white stripe at basal 1/3; head brown, vertex and labium black. Head smooth and impunctate. Pronotum transverse, 2.2 times wider than long, evenly convex on disc and lacking fovea or punctured depression, disc with scattered fine punctures; lateral margin rounded, anterior margin slightly concave, posterior margin straight. Elytra more or less widened posteriorly, apex convergently rounded, 1.4-1.5 times longer than wide, disc with punctures in part arranged in longitudinal rows, epipleurae smooth and impunctate.

**Figures 16–22.**
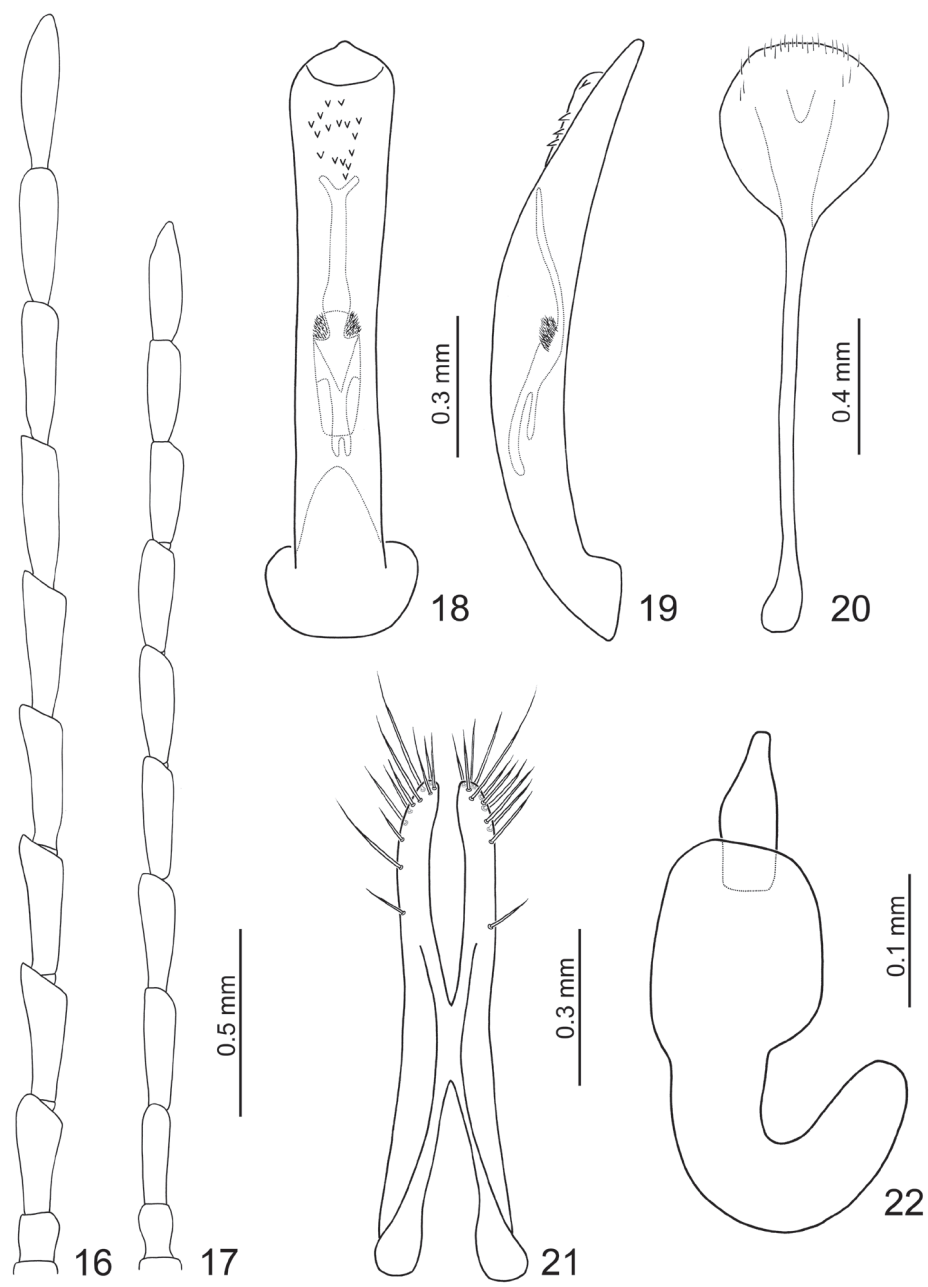
*Dercetina barclayi* sp. n. **16** Antenna, male **17** Antenna, female **18** Aedeagus, dorsal view **19** Aedeagus, lateral view **20** Sternite VIII **21** Gonocoxae **22** Spermatheca.

**Male**. Length 3.9–4.3 mm, width 2.1–2.4 mm. Antennomeres III-X weakly serrate (Fig. 16), ratio of length of antennomeres III to XI about 1.0: 1.2: 1.2: 1.2: 1.2: 1.2: 1.1: 1.1: 1.3; ratio of length to width of antennomeres III to XI about 2.6: 3.0: 3.3: 3.4: 3.4: 3.9: 3.8: 3.7: 4.8. Penis (Fig. 18) extremely slender, about 7.3 times longer than wide, parallel-sided, basally and apically widened, apex narrowly rounded; tectum membranous, with scatted stout setae; weakly curved in lateral view (Fig. 19); endophallic sclerites elongate, about 0.5 times as long as penis, dorsal sclerite with base bifurcate, apex truncate; ventral sclerite much longer than dorsal sclerite, apex bifurcate, base rounded, with a cluster of short setae at middle; in lateral view moderately curved.

**Female**. Length 4.7–5.3 mm, width 2.8–3.1 mm. Antenna 11-segmented, antennomeres III-X weakly serrate (Fig. 17), comparatively narrower than male, ratio of length of antennomeres III to XI about 1.0: 1.2: 1.2: 1.2: 1.2: 1.2: 1.0: 1.0: 1.2; ratio of length to width of antennomeres III to XI about 3.2: 3.6: 3.4: 3.6: 3.5: 3.3: 3.0: 3.2: 3.8. Sternite VIII (Fig. 20) weakly sclerotized subapically, apex rounded, with dense short setae along lateral and apical margin, spiculum long. Spermathecal receptaculum (Fig. 22) strongly swollen; pump narrow and strongly curved, apex widely rounded; spermathecal duct short, deeply projecting into receptaculum. Gonocoxae (Fig. 21) narrowly connected in middle, about 5.5 times longer than wide, slightly curved inwards near apex, apex rounded, with one short setae at apical 1/3, ten to eleven setae at apex.

#### Etymology.

This new species is named for Maxwell V. L. Barclay who is one of Britain’s leading entomologists and curator of Coleoptera at the Natural History Museum in London.

#### Distribution.

Only known from the type locality.

### Dercetina chinensis

(Weise, 1889) stat. r.

http://species-id.net/wiki/Dercetina_chinensis

Arthrotus chinensis Weise, 1889: 626; Ogloblin 1936: 330 (Jiangsu); Kimoto 1989b: 229 (as synonym of *Dercetina flavocincta* Hope, 1831)
Dercetina chinensis : Gressitt and Kimoto 1965: 802.
Antipha varipennis Jacoby, 1890: 214. synonymy confirmed
Dercetina varipennis : Gressitt and Kimoto 1963: 710 (as synonym of senior *Dercetina chinensis*); Kimoto 1965: 489; Gressitt and Kimoto 1965: 802 (corrected as junior synonym of *Dercetina chinensis*).

#### Type series.

*Arthrotus chinensis*: Holotype ♀ (MNHUB): “Yunnan Fischer / Yünnan Fischer / Arthrotus chinensis 89., m. / Typus (red label) / *Arthrotus chinensis* Wse L. N. Medvedev det. 1987”. Although locality labels of the holotype didn’t fit the original description, where Peking is clearly indicated, we prefer to treat it as true holotype because the specimen perfectly fit the original description and bears also original Weise´s handwritten identification label.

*Antipha varipennis*: Lecotype ♂ (BMNH), here designated, labeled: “Chang Yang A. E. Pratt Coll. July 1888. / Jacoby Coll. 1909-28a / *varipennis* Jac”. Paralectotypes: 1♂ (BMNH), same with lecotype but without determination label; 2♂♂ with black elytra and same labels are not designated as paralectotypes since Jacoby (1890) himself explicitely excluded dark specimens from the type series. Three more paralectotypes are deposited at the MCZC: 2♂♂, labeled “Chang Yang A. E. Pratt Coll. July 1888. / 1^st^ Jacoby Coll.”; 1♂, same but with additional labels: “Type 18269 (red label) / *Antipha varipennis* Jac. / Jan.-Jul. 2004 MCZ Image Database”.

#### Diagnosis.

*Dercetina chinensis* is similar to *Dercetina taiwana* by the absence of lateral fovea on the pronotum and by the antennomere IV being slightly longer than III. It can be distinguished by its characteristic color patterns (yellowish brown elytra with black longitudinal bands along suture and lateral margins) and oblong elytra (1.6 times longer than wide in contrast to 1.4 times longer than wide in *Dercetina taiwana*).

#### Redescription.

**Figures 23–28.**
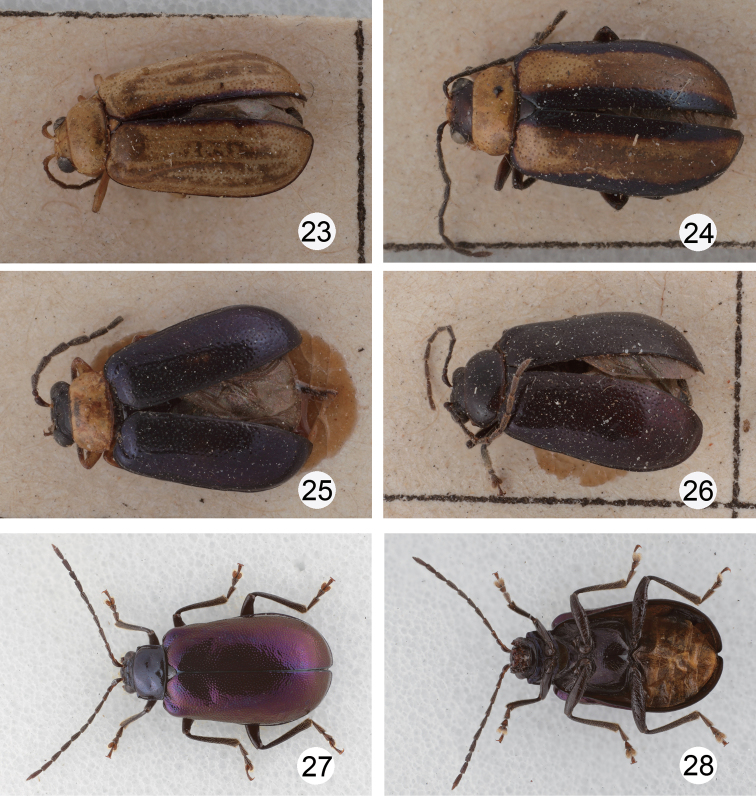
Habitus of *Dercetina* species. **23** *Dercetina chinensis*, pale individual **24** *Dercetina chinensis*, pale individual **25** *Dercetina chinensis*, dark individual **26** *Dercetina chinensis*, dark individual **27** *Dercetina itoi*, dorsal view **28** *Dercetina itoi*, ventral view.

Color very variable, in pale individuals generally yellowish brown, antennae, apical 2/3 of tibiae, and tarsi dark brown, meso- and metathoracic and abdominal ventrites blackish brown, margin of scutellum, and suture and lateral margins of elytra darkened (Fig. 23); in darker individuals black areas on elytra expanding inside, scutellum blackish brown, and head sometimes darkened (Fig. 24); in some individuals entire elytra black (Fig. 25); in darkest individuals entire body black (Fig. 26). Head smooth and impunctate. Pronotum transverse, 1.8 times wider than long, evenly convex on disc and lacking fovea or punctured depression, disc with scattered fine punctures; lateral margin rounded, anterior margin slightly concave, posterior margin slightly rounded. Elytra parallel-sided, apex convergently rounded, 1.6 times longer than wide, disc with random punctures, epipleurae smooth and impunctate.

**Figures 29–35.**
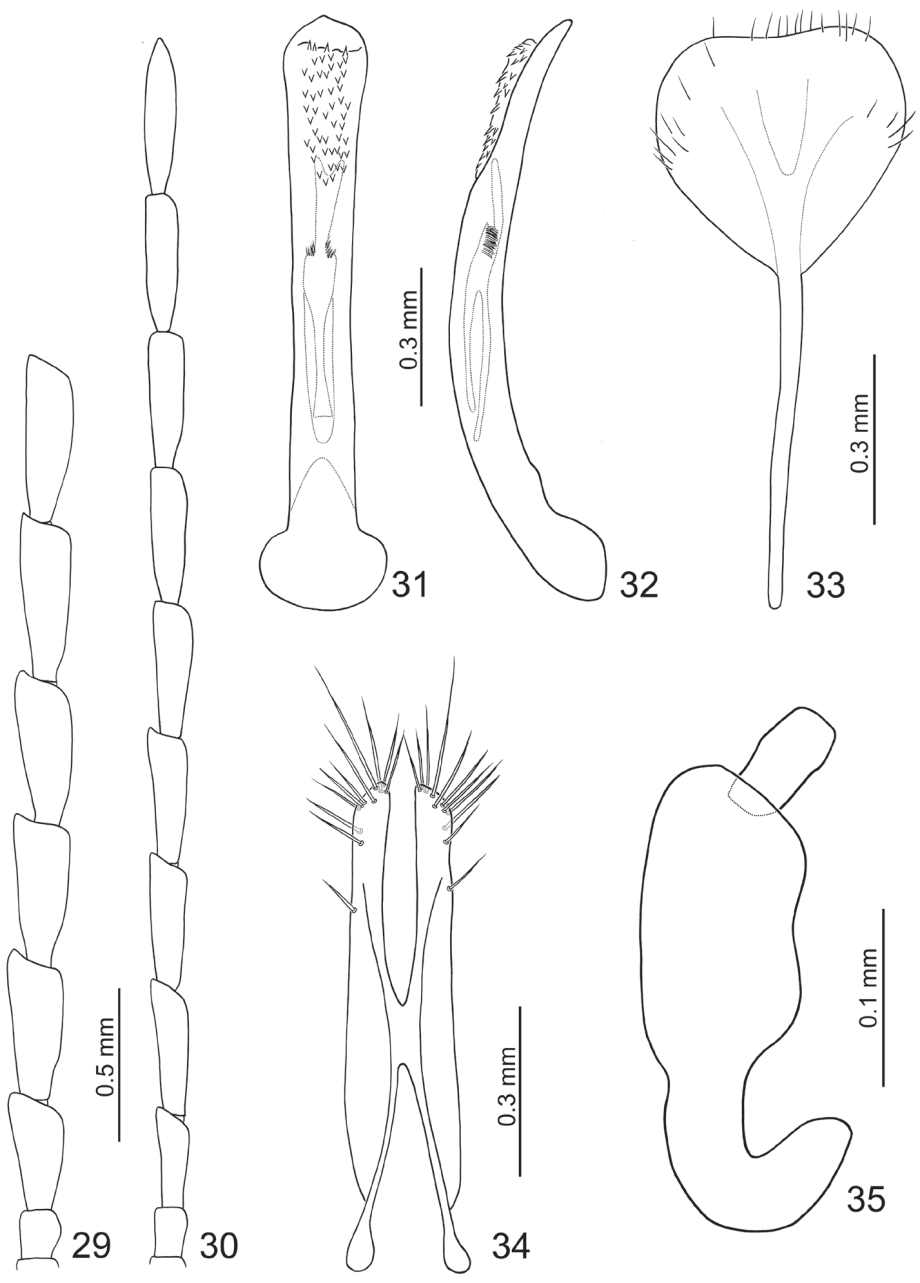
*Dercetina chinensis*. **29** Antenna, male **30** Antenna, female **31** Aedeagus, dorsal view **32** Aedeagus, lateral view **33** Sternite VIII **34** Gonocoxae **35** Spermatheca.

**Male.** Length 4.7–5.2 mm, width 2.4–2.8 mm. Atennomere II as long as antennomere III, ratio of length of antennomeres III to VIII (IX–XI lost) about 1.0: 1.3: 1.3: 1.3: 1.5: 1.5; ratio of length to width of antennomeres III to VIII about 2.3: 2.7: 2.9: 3.3: 3.5 (Fig. 29). Penis (Fig. 31) extremely slender, about 10.6 times longer than wide, parallel-sided, basally and apically widened; apex narrowly rounded, with small process in middle; tectum membranous, with dense stout setae; moderately curved in lateral view (Fig. 32); endophallic sclerites elongate, about 0.5 times as long as penis, apex concave and membranous, with a dorsal sclerite connected at middle, almost reaching base, with a row of short setae along lateral margin at apical 1/3; in lateral view almost straight.

**Female.** Length 4.5 mm, width 2.5 mm. Antenna comparatively narrower than male (Fig. 30), ratio of length of antennomeres III to XI about 1.0: 1.3: 1.3: 1.3: 1.3: 1.3: 1.3: 1.3: 1.6; ratio of length to width of antennomeres III to XI about 2.9: 3.4: 3.5: 3.5: 3.5: 4.0: 4.0: 4.0: 4.9. Sternite VIII (Fig. 33) very small, weakly sclerotized subapically, setae along lateral and apical margins, spiculum short. Spermathecal receptaculum (Fig. 35) strongly swollen; pump narrow and strongly curved, apex narrowly rounded; spermathecal duct short and stout, shallowly projecting into receptaculum. Gonocoxae (Fig. 34) narrowly connected in middle, elongate, about 4.7 times longer than wide, slightly curved inwards at apical 1/4, with one short setae at apical 1/3, ten short and long setae located apically or subapically.

#### Distribution.

China (Yunnan).

### Dercetina itoi

Kimoto, 1969

http://species-id.net/wiki/Dercetina_itoi

Dercetes flaviventris : Weise 1922: 94 (Taiwan; misidentification); Chûjô 1962: 143 (redescription).
Dercetina itoi Kimoto, 1969: 64 (Taiwan); Kimoto 1987: 190 (Taiwan).

#### Type series.

Holotype ♀ (KUEC): “(Taiwan) Alishan, 2300m Chiayi Hsien / 6.vii.1965 S. Ito / Japan-U. S. Co-op. Sci. Programme (yellow label) / HOLOTYPE (red label) / *Dercetina itoi* Kimoto, n. sp.”. Paratypes: 1 ex. (KMNH): “(Taiwan) Sungkang Nantou Hsien / 31.v.1965 T. Shirôzu / PARATYPE (blue label) / *Dercetina itoi* Kimoto, n. sp.”; 1 ex. (KMNH): “(Taiwan) Sungkang, 2000m – Tsuifeng, 2300m Nantou Hsien / 29.vi.1965 S. Kimoto / Japan-U. S. Co-op. Sci. Programme (yellow label) / PARATYPE (blue label) / *Dercetina itoi* Kimoto, n. sp.”; 1 ex. (KMNH): “[Formosa] Oiwake (Tsuifeng in Nantou county) 2,300 m 4.V.1965 T. Shirôzu / PARATYPE (blue label) / *Dercetina itoi* Kimoto, n. sp.”.

#### Material examined.

**TAIWAN:** 6♂♂, 4♀♀, Ilan, Ssuyuan, 25.IV.2009, leg. C.-F. Lee (TARI); 6♂♂, 12♀♀, same locality, 25.IV.2009, leg. M.-H. Tsou (TARI); 1♀, same locality, 9.VI.2009, leg. S.-F. Yu (TARI); 1♀, same locality, 6.V.2011, leg. S.-F. Yu (TARI); 1♀, Hohuanshan, 17.V.2009, leg. C.-F. Lee (TARI); 1♀, Nantou, Meifeng, 7–9.V.1981, leg. K. S. Lin & S. C. Lin (TARI); 1♀, same locality, 24–26.VI.1981, leg. K. S. Lin & W. S. Tang (TARI); 1♀, same locality, 22.V.1982, leg. L. Y. Chou (TARI); 1♀, same locality, 3.VII.2008, leg. M.-H. Tsou (TARI); 1♂, same locality, 20.IV.2011, leg. C.-F. Lee (TARI); 2♂♂, 2♀♀, same locality, 30.V.2011, leg. M.-H. Tsou (TARI); 1♀, Nantou, Tatachia, 9.VI.2009, leg. C.-F. Lee (TARI); 1♂, same locality, 20.VII.2009, leg. S.-F. Yu (TARI); 1♂, same locality, 21.IX.2009, leg. C.-F. Lee (TARI); 1♂, 4♀♀, same locality, 17.V.2010, leg. C.-F. Lee (TARI); 1♂, Nantou, Tsuifeng, 3.VI.1980, leg. L. Y. Chou & C. C. Chen (TARI); 2♂♂, 6♀♀, same locality, 8.V.1981, leg. K. S. Lin & S. C. Lin (TARI); 2♀♀, same locality, 25–27.VI.1981, leg. K. S. Lin & W. S. Tang (TARI); 1♂, 4♀♀, same locality, IV.1984, leg. K. S. Lin & K. C. Chou (TARI); 1♀, same locality, 23.VII.1984, leg. K. S. Lin (TARI); 2♂♂, 3♀♀, Nantou, Tungfu, 8.V.2011, leg. C.-F. Lee (TARI); 2♂♂, Nantou, Musha (= Wushe), 18.V.-15.VI.1919, leg. T. Okuni (TARI); 2♀♀, same locality, 6–11.V.1981, leg. K. S. Lin & S. C. Lin (TARI); 4♂♂, 3♀♀, Taichung, Pilu, 17.V.2009, leg. C.-F. Lee (TARI); 6♂♂, same locality, 17.V.2009, leg. M.-H. Tsou (TARI); 2♂♂, 1♀, Taoyuan, Lalashan, 1–2.IV.2009, leg. C.-F. Lee (TARI); 2♀♀, same locality, 14.V.2009, leg. C.-F. Lee (TARI).

#### Diagnosis.

*Dercetina itoi* is characterized by its metallic purple color. Some individuals of *Dercetina shirozui* with blackish brown legs are similar to *Dercetina itoi* in color pattern. *Dercetina itoi* can be separated from *Dercetina shirozui* by the tiny punctures on the pronotum (in contrast with the prominent puncures on the pronotum in *Dercetina shirozui*).

#### Redescription.

Color (Figs 3–4) dark metallic purple, antennae and legs blackish brown, abdomen yellowish brown. Antennal calli separated by deep furrow; vertex with distinct punctures, disc shagreened. Pronotum 1.6–1.7 times wider than long, disc evenly convex, with scattered fine punctures, and a pair of deep round fovea at sides; lateral margin straight, narrowed posteriorly, anterior margin straight, posterior margin slightly rounded. Elytra more or less widened posteriorly, apex convergently rounded, 1.6–1.7 times longer than wide, disc with densely prominent punctures; epipleurae impunctate, somewhat rugose.

**Figures 36–42.**
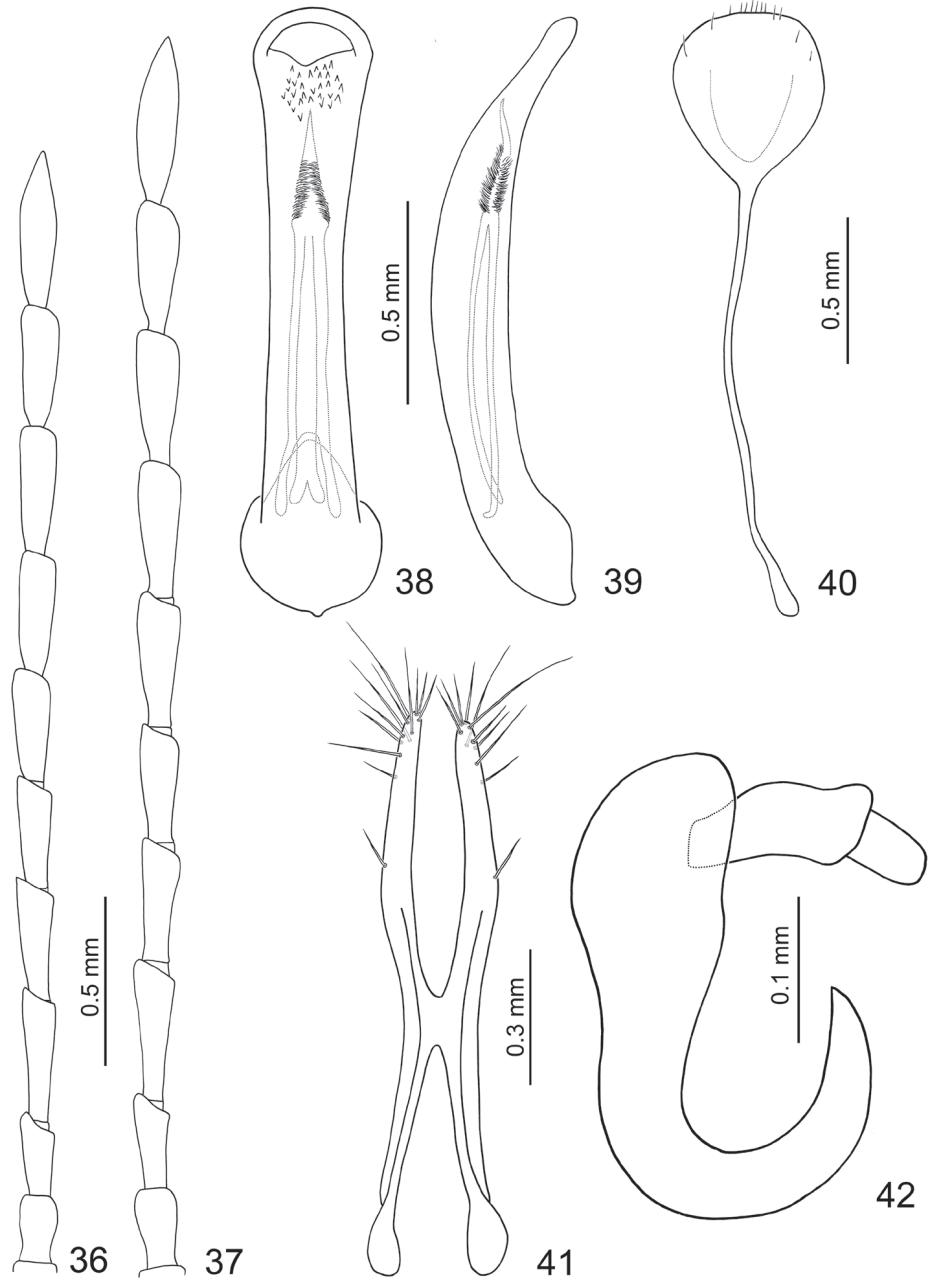
*Dercetina itoi*. **36** Antenna, male **37** Antenna, female **38** Aedeagus, dorsal view **39** Aedeagus, lateral view **40** Sternite VIII **41** Gonocoxae **42** Spermatheca.

**Male.** Length 5.1–5.3 mm, width 2.4–2.6 mm., Antennomeres VIII-XI filiform, ratio of length of antennomeres III to XI about 1.0: 1.5: 1.4: 1.3: 1.4: 1.5: 1.6: 1.5: 1.9; ratio of length to width of antennomeres III to XI about 2.4: 3.6: 3.0: 2.7: 2.9: 3.5: 3.7: 3.5: 4.0 (Fig. 36). Penis (Fig. 38) slender, about 7.7 times longer than wide, parallel-sided, apically and basally widened, apex broadly rounded; tectum membranous, with scattered stout setae; weakly curved in lateral view (Fig. 39); endophallic sclerite elongate, about 0.7 times as long as penis, apex pointed, and recurved in lateral view, with a cluster of setae near apex; dorsal sclerite with base deeply bifurcate, narrower than ventral sclerite, base bifurcate, in lateral view weakly curved.

**Female.** Length 5.9–6.6 mm, width 3.0–3.3 mm. Ratio of length of antennomeres III to XI about 1.0: 1.4: 1.3: 1.3: 1.4: 1.4: 1.4: 1.3: 1.8; ratio of length to width of antennomeres III to XI about 2.7: 3.3: 3.2: 3.0: 3.2: 3.1: 3.2: 3.1: 4.1 (Fig. 37). Sternite VIII (Fig. 40) weakly sclerotized subapically, apex truncate, with few setae along apical margin, spiculum extremely long. Spermathecal receptaculum (Fig. 42) weakly swollen; pump narrow and strongly curved, apex pointed; spermathecal duct long, deeply projecting into receptaculum. Gonocoxae (Fig. 41) narrowly connected at middle, about 4.8 times longer than wide, curved inwards at apical 1/3, with one short setae at apical 1/3, ten or eleven setae at apex.

#### Host plants.

Sabiaceae: *Sabia transarisanensis* Hayata; Stachyuraceae: *Stachyurus himalaicus* Hook. f. & Thomson ex Benth.

#### Distribution.

Taiwan. It is widespread in high mountains (above 2000 m) (Fig. 13).

### Dercetina shirozui

Kimoto, 1969

http://species-id.net/wiki/Dercetina_shirozui

Dercetina shirozui Kimoto, 1969: 63; Kimoto 1989b: 260 (Taiwan); Kimoto 1991: 17 (Taiwan).

#### Type series.

Holotype ♀ (KUEC): “(Taiwan) Sungkang Nantou Hsien / 5.V.1965 T. Shirôzu / HOLOTYPE (red label) / *Dercetina shirozui* Kimoto, n. sp.”. Paratypes: 1 ex., same as holotype (KMNH); 1 ex.: “(Taiwan) Sungkang Nantou Hsien / 1.VI.1965 T. Shirôzu / PARATYPE (blue label) / *Dercetina shirozui* Kimoto, n. sp.” (KMNH); 1 ex.: “(Taiwan) Sungkang Nantou Hsien / 18.V.1965 T. Shirôzu / PARATYPE (blue label) / *Dercetina shirozui* Kimoto, n. sp.” (KMNH); 1 ex.: “(Taiwan) Taiko (= Tahu) – Nihonmatsu (= Sungen) Byoritsu-ken (= Miaoli county) 9.iv.1967 T. Shirozu / PARATYPE (blue label) / *Dercetina shirozui* Kimoto, n. sp.” (KMNH); 1 ex.: “(Taiwan) Alishan, 2300m Chiayi Hsien / 6.vii.1965 S. Kimoto / Japan-U. S. Co-op. Sci. Programme (yellow label) / PARATYPE (blue label) / *Dercetina shirozui* Kimoto, n. sp.” (KMNH); 1 ex.: “(Taiwan) Hokuko (= Peikeng) – Kaminoshima-onsen (= Hushan) Byoritsu-ken (= Miaoli county) 11.iv.1967 T. Shirozu / PARATYPE (blue label) / *Dercetina shirozui* Kimoto, n. sp.” (KMNH).

#### Material examined.

**TAIWAN**: 1♂, Hsinchu, Litungshan, 23.III.2007, leg. M.-H. Tsou (TARI); 3♂♂, same locality, 15.III.2009, leg. M.-H. Tsou (TARI); 1♂, Hsinchu, Mamei, 13.III.2011, leg. M.-H. Tsou (TARI); 2♀♀, Hsinchu, Tahunshan, 24.II.2009, leg. S.-F. Yu (TARI); 1♂, 2♀♀, same locality, 1.III.2009, leg. M.-H. Tsou (TARI); 1♂, Hsinchu, Wufeng, 17.II.2009, leg. S.-F. Yu (TARI); 1♂, Hualien, Pilu, 17.V.2009, leg. C.-F. Lee (TARI); 8♂♂, Ilan, Chiuchihtse, 7.XII.2008, leg. M.-H. Tsou (TARI); 1♂, 1♀, Ilan, Suyuan, 28.IV.2009, leg. M.-H. Tsou (TARI); 1♀, Kaoshiung, Tengchih (= Shihshan trail), 2–5.VI.2008, leg. C.-F. Lee (TARI); 22♂♂, same locality, 6.II.2009, leg. M.-H. Tsou (TARI); 1♀, Nantou, Hohuanshan, 18.V.2009, leg. C.-F. Lee (TARI); 1♀, Nantou, Meifeng, 5–8.VI.1980, leg. C. C. Chen (TARI); 1♀, same locality, 7–9.V.1981, leg. K. S. Lin & S. C. Lin (TARI); 1♀, same locality, 24–26.VI.1981, leg. K. S. Lin & W. S. Tang (TARI); 1♂, 1♀, same locality, 22.V.1982, leg. L. Y. Chou (TARI); 1♀, Nantou, Tattaka (= Sungkang), VI.1925, leg. J. Sonan (TARI); 1♂, 1♀, same locality, 4.IV.2010, leg. Y.-T. Wang (TARI); 2♂♂, Nantou, Tatachia, 9.VI.2009, leg. C.-F. Lee (TARI); 3♀♀, same locality, 27.IV.2010, leg. C.-F. Lee (TARI); 2♀♀, Nantou, Tayuling, 9-16.VI.1981, leg. K. S. Lin & B. H. Chen (TARI); 1♀, Nantou, Tsuifeng, 3.VI.1980, leg. L. Y. Chou & C. C. Chen (TARI); 3♀♀, same locality, 8.V.1981, leg. K. S. Lin & S. C. Lin (TARI); 1♀, same locality, 25–27.VI.1981, leg. K. S. Lin & W. S. Tang (TARI); 2♀♀, Nantou, Wushe, 6–11.V.1981, leg. K. S. Lin & S. C. Lin (TARI); 1♂, Pingtung, Tahanshan, 2.II.2008, leg. M.-H. Tsou (TARI); 1♀, same locality, 3.III.2008, leg. C.-F. Lee (TARI); 1♂, 1♀, same locality, 21.III.2009, leg. M.-H. Tsou (TARI); 1♂, same locality, 6.III.2010, leg. U. Ong (TARI); 1♂, same locality, 28.IV.2012, leg. M.-H. Tsou (TARI); 2♀♀, same locality, 16.IV.2012, leg. C.-F. Lee (TARI); 1♂, 2♀♀, Taoyuan, Hsitsun, 12.IV.2009, leg. M.-H. Tsou (TARI); 2♂♂, 2♀♀, Taoyuan, Hsuehwunao, 10.IV.2011, leg. M.-H. Tsou (TARI); 1♀, Taoyuan, Lalashan, 2.IV.2009, leg. H.-J. Chen (TARI); 1♀, same locality, 2.IV.2009, leg. C.-F. Lee (TARI); 1♂, same locality, 4.V.2010, leg. S.-F. Yu (TARI); 1♀, same locality, 14.V.2009, leg. C.-F. Lee (TARI).

#### Diagnosis.

*Dercetina shirozui* is similar to *Dercetina azumai* with metallic green elytra and yellowish brown legs but differs by its metallic green head, prothorax, meso- and metathoracic ventrites (in contrast with yellowish brown ones in *Dercetina azumai*). Some individuals of *Dercetina shirozui* with blackish brown legs may be similar to *Dercetina itoi*. However, *Dercetina shirozui* is distinguished from *Dercetina itoi* by the prominent punctures on the pronotum.

#### Redescription.

**Figures 43–48.**
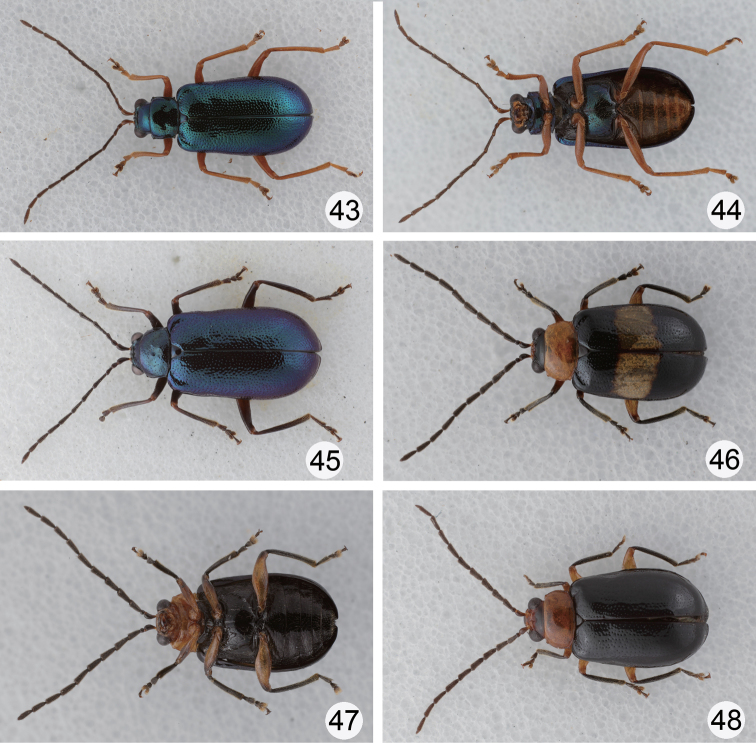
Habitus of *Dercetina* species. **43** *Dercetina shirozui*, dorsal view **44** *Dercetina shirozui*, ventral view **45** *Dercetina shirozui*, color variation **46** *Dercetina taiwana*, dorsal view **47** *Dercetina taiwana*, ventral view **48** *Dercetina taiwana*, color variation.

Color (Figs 43–44) metallic green or blue, legs and abdomen yellowish brown, antenna dark brown or blackish brown. Antennal calli with deep furrow in middle; vertex with dense distinct punctures, disc shagreened. Pronotum 1.5-1.6 times wider than long, disc evenly flat, with dense prominent punctures, and a pair of deep round fovea at sides; lateral margin straight, narrowed posteriorly, anterior margin slightly concave, posterior margin slightly rounded. Elytra more or less widened posteriorly, apex convergently rounded, 1.7–1.8 times longer than wide, disc with densely prominent punctures; epipleurae with dense prominent punctures.

**Figures 49–55.**
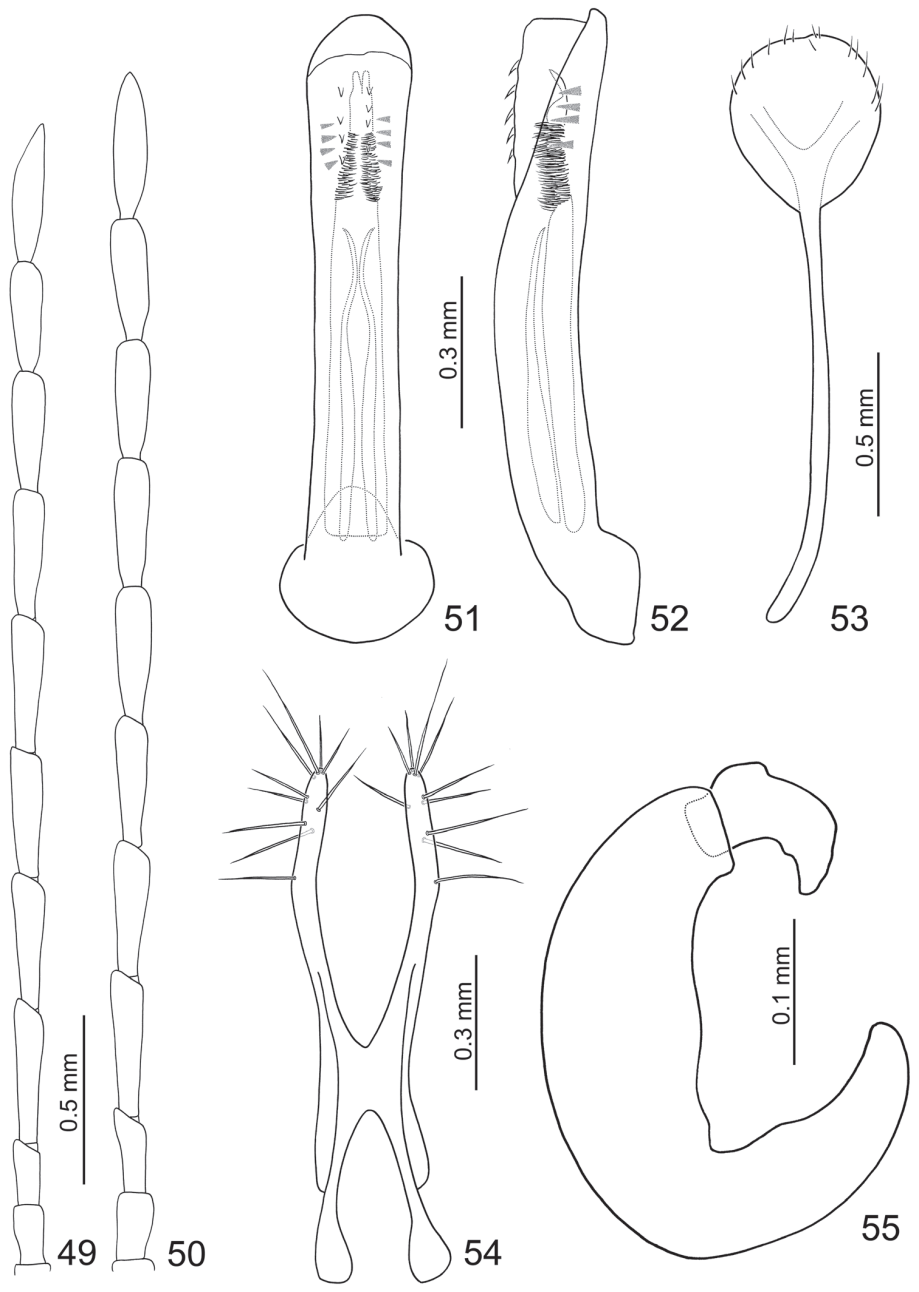
*Dercetina shirozui*. **49** Antenna, male **50** Antenna, female **51** Aedeagus, dorsal view **52** Aedeagus, lateral view **53** Sternite VIII **54** Gonocoxae **55** Spermatheca.

**Male.** Length 4.8–5.1 mm, width 2.1–2.3 mm. Ratio of length of antennomeres III to XI about 1.0: 2.2: 2.0: 2.0: 2.1: 2.0: 1.9: 1.7: 2.2; ratio of length to width of antennomeres III to XI about 2.4: 4.9: 3.9: 4.1: 4.1: 3.9: 3.8: 3.4: 4.6 (Fig. 49). Penis (Fig. 51) slender, about 7.3 times longer than wide, parallel-sided, basally widened, apex broadly rounded; tectum membranous, with two longitudinal rows of stout setae; weakly curved in lateral view (Fig. 52); endophallus with four pairs of small teeth near apex, ventral sclerite long, about 0.7 times as long as penis, base truncate, apex bifurcate and curved in lateral view; with a cluster of setae near apex; a pair of elongate dorsal sclerites located from base to apical 1/3, apices pointed and recurved subapically.

**Female.** Length 5.5–6.3 mm, width 2.7–3.0 mm. Antenna 11-segmented, filiform (Fig. 50), ratio of length of antennomeres III to XI about 1.0: 1.6: 1.5: 1.5: 1.5: 1.5: 1.4: 1.4: 1.7; ratio of length to width of antennomeres III to XI about 2.6: 4.3: 3.7: 3.7: 3.6: 3.7: 3.4: 3.4: 4.1. Sternite VIII (Fig. 53) weakly sclerotized laterally and apically, with a few setae along apical margin, spiculum extremely long. Spermathecal receptaculum (Fig. 51) elongate and weakly swollen; pump narrow and strongly curved, apex narrowly rounded; spermathecal duct short, apically narrowed, shallowly projecting into receptaculum. Gonocoxae (Fig. 54) widely connected in middle, about 3.4 times longer than wide, curved inwards in apical 1/3, with ten long setae in apical 1/3.

**Color variation.** Some populations have blackish brown legs and black antenna (Fig. 45).

#### Host plants.

Aceraceae: *Acer albopurpurascens* Hayata; Actinidiaceae: *Actinidia callosa* Lindl.; Rosaceae: *Prunus phaeosticta* (Hance) Maxim.; Saxifragaceae: *Deutzia pulchra* Vidal and *Schizophragma integrifolium* Oliv. var. *fauriei* (Hayata) Hayata; Stachyuraceae: *Stachyurus himalaicus* Hook. f. & Thomson ex Benth.; Staphyleaceae: *Turpinia formosana* Nakai.

#### Distribution.

Taiwan. It is widespread in mountains above 1000 m (Fig. 14).

### Dercetina taiwana

(Chûjô, 1938) stat. r.

http://species-id.net/wiki/Dercetina_taiwana

Dercetis taiwana Chûjô, 1938: 140; Chûjô 1962: 145 (redescription); Gressitt and Kimoto 1963: 710 (as synonymy of *Dercetina chinensis*).
Dercetis taiwana ab. *melania* Chûjô, 1938: 140; Gressitt and Kimoto 1963: 710 (confirmed as infraspecific variation of *Dercetina chinensis*)
Dercetis taiwana var. *melania*: Chûjô 1962: 146 (redescription).
Dercetis taiwana f. *melania*: Chûjô 1965: 95 (Taiwan).
Dercetina chinensis : Kimoto 1966: 33 (Taiwan); Kimoto 1969: 66 (Taiwan); Takizawa et al. 1995: 63 (Taiwan).
Dercetina flavocincta : Kimoto 1989a: 260 (Taiwan); Kimoto 1991: 17 (Taiwan).

#### Type series.

*Dercetis taiwana*: Lecotype ♂, here designated, labeled: “Formosa Shinchiku (= Hsinchu), -18. VII 1–30. J. Sonan / COType (circular label with yellow letters and border) / *Dercetis taiwana* CHÛJÔ DET. M. CHUJO / 1916” (TARI). Paralectotypes: 3♀♀: “Urai (= Wulai, in Taipei county) FORMOSA 28.III.1932 COL. M. CHUJO / COType (circular label with yellow letters and border) / *Dercetis taiwana* CHÛJÔ DET. M. CHUJO / 2379, 2380, 2384 (respectively)” (TARI); 2♀♀: “Taiheizan (= Taipingshan, in Ilan county) FORMOSA Jul 1930 S. Minowa / COType (circular label with yellow letters and border) / *Dercetis taiwana* CHÛJÔ DET. M. CHUJO / 2162 or 2385” (TARI); 1♀: “Formosa Arisan (= Alisan, in Chiayi county), 1918 X 2-23. J. Sonan COType (circular label with yellow letters and border) / *Dercetis taiwana* CHÛJÔ DET. M. CHUJO / 2159” (TARI); 1♂: “Formosa Y. Miwa / Rimogan (= Fushan, in Taipei county) 22.7.1929 (on the back) / COType (circular label with yellow letters and border) / *Dercetis taiwana* CHÛJÔ DET. M. CHUJO / 2160” (TARI); 1♀: “Formosa Y. Miwa / Kobayashi, 25.7.1929 / Syntypus (red label) / *Dercetis taiwana* CHÛJÔ DET. M. CHUJO / DEI Müncheberg Col - 03095” (SDEI); 1♀: “Paroe (Form.) H. Sauter IX.1912 / Syntypus (red label) / *Dercetis taiwana* CHÛJÔ DET. M. CHUJO / DEI Müncheberg Col - 03096” (SDEI); 1♀: “Taihorin (= Talin, in Chiayi county) Formosa H. Sauter, 1911 / 7.VII. / Syntypus (red label) / *Dercetis taiwana* CHÛJÔ DET. M. CHUJO / DEI Müncheberg Col - 03097” (SDEI); 1♀: “Formosa Y. Miwa / Hsuanyan (in Japanese) 23.7.1929 / COType (circular label with yellow letters and border) / *Dercetis taiwana* CHÛJÔ DET. M. CHUJO / 2161” (TARI).

*Dercetis taiwana* ab. *melania*: Syntypes: 1♂, 1♀: “Urai (= Wulai, in Taipei county) FORMOSA 28.III.1932 COL. M. CHUJO / COType (circular label with yellow letters and border) / *Dercetis taiwana* ab. *melania* CHÛJÔ DET. M. CHUJO / 2374 or 2375” (TARI); 2♀♀: “Urai (= Wulai, in Taipei county) FORMOSA 28.III.1932 COL. M. CHUJO / Syntypus (red label) / *Dercetis taiwana* ab. *melania* CHÛJÔ DET. M. CHUJO / DEI Müncheberg Col – 03098 and 03099” (SDEI); 1♂, 1♀: “Formosa Shinchiku (= Hsinchu), -18. VII 1-30. J. Sonan / COType (circular label with yellow letters and border) / *Dercetis taiwana* ab. *melania* CHÛJÔ DET. M. CHUJO / 2163 or 1923” (TARI); 1♀: “Formosa Y. Miwa / Urai (= Wulai in Taipei county) 20.7.1929 (on the back) / COType (circular label with yellow letters and border) / *Dercetis taiwana* ab. *melania* CHÛJÔ DET. M. CHUJO / 1915” (TARI); 1♂: “Formosa Y. Miwa / Hsitsun (in Chinensis) 24.7.1929 (on the back) / COType (circular label with yellow letters and border) / *Dercetis taiwana* ab. *melania* CHÛJÔ DET. M. CHUJO / 2164”.

#### Material examined.

**CHINA**: 1♂, 1♀, Guangdong, Tsha-jiu-san, V.-VI.1912, leg. S. V. Mell (KMNH); 1♂, Hubei, Lichuan, Suisapa, 23.VIII.1948, leg. J. L. Gressitt (KMNH); 1♂, Hubei, Lichuan, Lianghokeu, 1.IX.1948, leg. Gressitt & Djou (KMNH); **TAIWAN**: 1♀, Hsinchu, Chienshih, 26.VII.2008, leg. H.-J. Chen (TARI); 1♂, same locality, 26.IX.2009, leg. H.-J. Chen (TARI); 1♂, 1♀, Hsinchu, Lupi, 24.VI.2008, leg. H. Lee (TARI); 1♀, Hsinchu, Peitelaman, 26.VI.2008, leg. S.-F. Yu (TARI); 3♂♂, 5♀♀, Hsinchu, Talu trail, 22.VIII.2009, leg. Y.-L. Lin (TARI); 1♀, same locality, 19.VI.2010, leg. Y.-L. Lin (TARI); 2♂♂, Hsinchu, Wufeng, 12.VII.2008, leg. H.-J. Chen (TARI); 1♀, same locality, 29.IX.2009, leg. Y.-L. Lin (TARI); 1♂, 2♀♀, Hsinchu, Yulao, 3.IV.2011, leg. M.-H. Tsou (TARI); 2♀♀, Ilan, Fushan Botanical Park, 1.IV.2008, leg. H.-J. Chen (TARI); 4♂♂, 5♀♀, same locality, 1.IV.2008, leg. M.-H. Tsou (TARI); 1♂, same locality, 2.IV.2008, leg. H.-J. Chen (TARI); 2♂♂, same locality, 20.III.2009, leg. C.-F. Lee (TARI); 1♂, 1♀, Ilan, Mingchi, 17.III.2007, leg. M.-H. Tsou (TARI); 2♂♂, 7♀♀, same locality, 29.VII.2007, leg. M.-H. Tsou (TARI); 3♂♂, 2♀♀, same locality, 27.IV.2008, leg. S.-F. Yu (TARI); 2♀♀, same locality, 25.V.2008, leg. M.-H. Tsou (TARI); 1♀, Ilan, Suchi trail, 19.V.1910, leg. H.-J. Chen (TARI); 1♀, Ilan, Taipingshan, 3.VI.2007, leg. S.-F. Yu (TARI); 1♂. 5♀♀, Ilan, Tsuifenghu, 3.VII.2010, leg. M.-H. Tsou (TARI); 1♀, Taichung, Yuantsuishan, 16.VII.2010, leg. J.-C. Chen (TARI); 2♀♀, Taipei, Chutzuhu, 16.IX.2007, leg. S.-F. Yu (TARI); 3♂♂, same locality, 15.VI.2008, leg. M.-H. Tsou (TARI); 1♂, 1♀, Taipei, Erhkoshan, 26.XI.2006, leg. M.-H. Tsou (TARI); 1♂, 1♀, Taipei, Fengkueitsui, 29.VI.2007, leg. S.-F. Yu (TARI); 1♀, Taipei, Fushan, 5.IV.2007, leg. S.-F. Yu (TARI); 1♂, 2♀♀, Taipei, Hsiaoyuken, 29.III.2008, leg. M.-H. Tsou (TARI); 1♂, 2♀♀, Taipei, Lengshuiken, 22.VI.2008, leg. S.-F. Yu (TARI); 3♂♂, 2♀♀, Taipei, Menghu, 27.IX.2007, leg. S.-F. Yu (TARI); 3♂♂, 7♀♀, Taipei, Pinglin, 29.III.2008, leg. M.-H. Tsou (TARI); 1♂, 3♀♀, Taipei, Wulai, 10.VII.2007, leg. H.-T. Cheng (TARI); 4♀♀, same locality, 27.IX.2006, leg. H.-J. Chen (TARI); 2♂♂, 3♀♀, same locality, 22.X.2006, leg. S.-F. Yu (TARI); 1♂, 1♀, same locality, 26.X.2006, leg. S.-F. Yu (TARI); 2♀♀, same locality, 19.VI.2007, leg. M.-H. Tsou (TARI); 2♀♀, 30.IX.2007, leg. M.-H. Tsou (TARI); 1♀, Taipei, Yangmingshan, 3.III.1998, leg. C.-F. Lee (TARI); 1♂, same locality, 15.III.1998, leg. C.-F. Lee (TARI); 1♂, 1♀, same locality, 29.VIII.2006, leg. H.-J. Chen (TARI); 1♀, same locality, 15.IV.2007, leg. M.-H. Tsou (TARI); 1♀, same locality, 19.V.2007, leg. M.-H. Tsou (TARI); 2♀♀, same locality, 1.VI.2007, leg. S.-F. Yu (TARI); 3♂♂, 1♀, same locality, 23.VI.2007, leg. S.-F. Yu (TARI); 1♂, Taipei, Yingtzuling, 24.VII.2010, leg. Y.-L. Lin (TARI); 2♀♀, same locality, 25.IX.2010, leg. Y.-L. Lin (TARI); 1♀, Taoyuan, Hsiaowulai, 29.IX.2009, leg. M.-H. Tsou (TARI); 1♂, same locality, 1.VI.2010, leg. S.-F. Yu (TARI); 1♂, Taoyuan, Tsuehwunao, 10.IV.2011, leg. M.-H. Tsou (TARI); 2♂♂, Taoyuan, Mamei, 3.IV.2011, leg. M.-H. Tsou (TARI); 6♂♂, 13♀♀, Taoyuan, Paling, 8.XI.2009, leg. M.-H. Tsou (TARI); 1♂, same locality, 11.IV.2010, leg. M.-H. Tsou (TARI); 1♂, 1♀, Taoyuan, Tungyanshan, 14.VII.2008, leg. H. Lee (TARI); 1♀, same locality, 17.IV.2009, leg. H. Lee (TARI); 3♂♂, same locality, 10.V.2009, leg. H. Lee (TARI); 1♀, same locality, 16.VI.2009, S.-F. Yu (TARI);

#### Diagnosis.

*Dercetina taiwana* is similar to *Dercetina chinensis* and *Dercetina barclayi* sp. n. in the absence of lateral fovea on the pronotum and antennomere III slightly longer than IV. *Dercetina taiwana* and *Dercetina chinensis* are separated from *Dercetina barclayi* sp. n. by its sexually dimorphic antennae. Both species have their distinct color patterns in most individuals: the bluish black elytra with white transverse band at middle in *Dercetina taiwana*; the yellowish brown elytra with or without longitudinal black bands long suture and lateral margins in *Dercetina chinensis*. Although some individual specimens of both species share the same color pattern, *Dercetina taiwana* differs from *Dercetina chinensis* by a more oval elytra (1.4 times longer than wide in contrast with 1.6 times longer than wide in *Dercetina chinensis*).

#### Redescription.

Color (Figs 46–47) bluish black, head (except eyes and antennae), prothorax, coxae, and tibiae yellowish brown; elytra with one transverse white stripe at basal 1/3. Head smooth and impunctate. Pronotum transverse, 1.9–2.0 times wider than long, evenly convex on disc and lacking fovea or punctured depression, disc with scattered fine punctures; lateral margin rounded, anterior margin concave, posterior margin slightly rounded. Elytra more or less widened posteriorly, apex convergently rounded, 1.4 times longer than wide, disc with punctures in part arranged in longitudinal rows, epipleurae smooth and impunctate.

**Figures 56–62.**
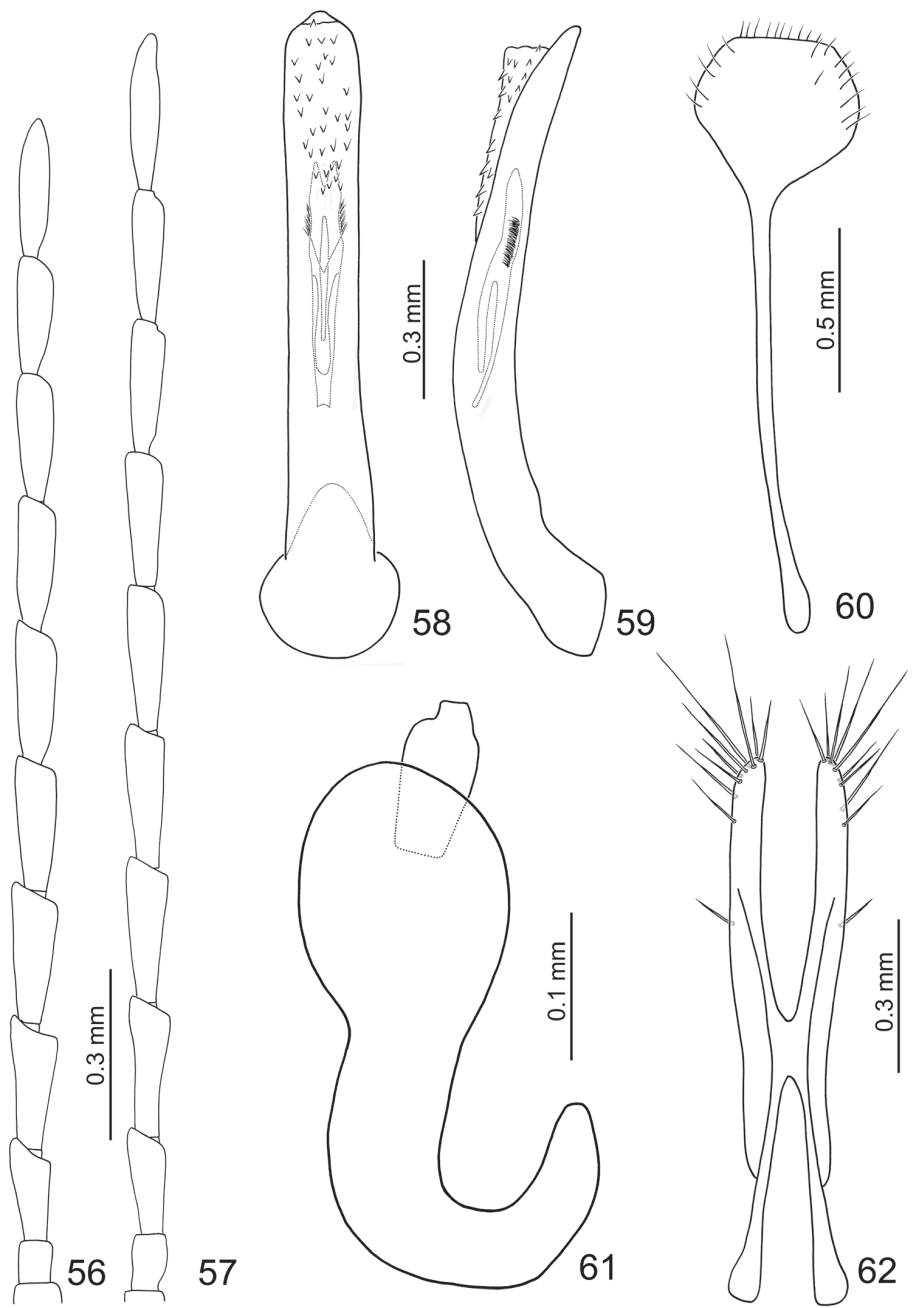
*Dercetina taiwana*. **56** Antenna, male **57** Antenna, female **58** Aedeagus, dorsal view **59** Aedeagus, lateral view **60** Sternite VIII **61** Spermatheca **62** Gonocoxae.

**Male.** Length 4.0–4.5 mm, width 2.2–2.5 mm. Ratio of length of antennomeres III to XI about 1.0: 1.3: 1.3: 1.3: 1.3: 1.2: 1.2: 1.1: 1.3; ratio of length to width of antennomeres III to XI about 2.5: 2.9: 3.0: 3.2: 3.3: 3.3: 3.6: 3.6: 4.5 (Fig. 56). Penis (Fig. 58) extremely slender, about 8.4 times longer than wide, parallel-sided, basally widened, apex narrowly rounded; tectum membranous, with scatted stout setae; weakly curved in lateral view (Fig. 59); endophallic sclerite elongate, about 0.4 times as long as penis, base and apex concave and membranous, with a dorsal sclerite connected at basal 1/4, almost reaching base, with a row of short setae along lateral margin at apical 1/4; in lateral view almost straight.

**Female.** Length 5.2–5.7 mm, width 3.0–3.3 mm. Antenna 11-segmented, filiform (Fig. 57), comparatively narrower than in male, ratio of length of antennomeres III to XI about 1.0: 1.3: 1.3: 1.3: 1.3: 1.3: 1.3: 1.2: 1.5; ratio of length to width of antennomeres III to XI about 2.9: 3.1: 3.3: 3.5: 3.6: 3.5: 3.9: 4.0: 5.4. Sternite VIII (Fig. 60) weakly sclerotized subapically, apex truncate, setae along lateral and apical margin, spiculum long. Spermathecal receptaculum (Fig. 61) swollen; pump narrow and strongly curved, apex narrowly rounded; spermathecal duct short, deeply projecting into receptaculum. Gonocoxae (Fig. 62) narrowly connected in middle, about 4.6 times longer than wide, straight, with one short setae at apical 1/3 and nine setae at apex.

**Color variation.** A number of individuals have transverse white strip on elytra reduced at various degrees, or even totally absent (Fig. 48); some have the vertex, clypeus, and labrum darkened; a few individuals have pronotum darkened.

#### Host plants.

Betulaceae: *Alnus formosana* (Burkill ex Forbes & Hemsl.) Makin; Compositae: *Eupatorium formosanum* Hayata; Fagaceae: *Quercus variabilis* Bl.; Lauraceae: *Cinnamomum camphora* (L.) Presl. and *Litsea cubeba* (Lour.) Persoon; Lythraceae: *Lagerstroemia subcostata* Koehne; Moraceae: *Humulus scandens* (Lour.) Merr.; Rosaceae: *Prunus campanulata* Maxim.; Rutaceae: *Tetradium glabrifolium* (Champ. ex Benth.) T. Hartley; Stachyuraceae: *Stachyurus himalaicus* Hook. f. & Thomson ex Benth.; Ulmaceae: *Trema orientalis* (L.) Bl.

#### Distribution.

China (Guandung, Hubei) and Taiwan. *Dercetina taiwana* is widespread in northern Taiwan from lowlands to high mountains (above 2000 m) (Fig. 15). This species is rather abundant.

### Dercetina unifasciata

(Allard, 1889) stat. r. and comb. n.

http://species-id.net/wiki/Dercetina_unifasciata

Antipha unifasciata Allard, 1889: 107; Kimoto 1989b: 229. (as synonymy of *Dercetina flavocincta*)

#### Type material.

*Antipha unifasciata*: Holotype ♀ (head lost; ISNB): “Cambodge / Coll. Chapuis / E. Allard det.: *Antipha unifasciata* All. / *Antipha unifasciata* All. (yellow label) / TYPE (pink label) / cf. Ann. Soc Ent. Belg., XXXIII, 1889, p. 107-108 / sec. Weise, Col. Cat. Junk (78), 1924: p. 145*Dercetis unifasciata* All.”.

#### Material examined.

**CAMBODIA**: 1♀, Kampot prov., Bokor, 8.V.1961, leg. J. L. Nickel (USNM); **INDIA**: 1♀ (BMNH), Darjeeling, Bengal, Debrepani, 6000’, 18.IX.1929, leg. J.C.M. Gardner; **LAOS**: 1♀, Attapeu Prov., Houei Kong, 31.V.1965, leg. Native Collector (BPBM); 1♀, Borikhane Prov., Pakkading, 31.VII.1965, leg. Native Collector (BPBM); 1♀, Sayabouly Prov., Sayaboury, 16.IV.1965, leg. J. L. Gressitt (BPBM); 1♀, Sedone Prov., Paksong, 18.V.1965, leg. P. D. Ashlock (BPBM); 1♂, 1♀, Vientiane Prov., Ban Van Eue, 20km E of Phou-kow-kuei, 1–15.V.1965, leg. J. A. Rondon (BPBM); 1♂, same locality, 11.IV.1965, leg. J. L. Gressitt (KMNH); 1♂, 1♀, same locality, 12.IV.1965, leg. J. L. Gressitt (BPBM); 1♂, 15.VIII.1965, leg. J. L. Gressitt (BPBM); 1♀, same locality, 15.III.1966, leg. Native Collector (BPBM); 1♀, 15.XII.1966, leg. Native Collector (BPBM); 1♀, same locality, 2.II.1968, leg. Native Collector (BPBM); 1♀, same locality, 15.II.1968, leg. Native Collector (BPBM); 1♀, same locality, 15.VIII.1968, leg. Native Collector (BPBM); 1♀, Phou-kow-kuei, 15.IV.1965, leg. J. L. Gressitt (BPBM); 1♀, Vientiane Prov., Vientiane, 30.IV.1967, leg. Native Collector (BPBM); 1♀, Xieng Kyouang Prov., Ban Sam Thang, 5.XI.1965, leg. Native Collector (BPBM).

#### Diagnosis.

*Dercetina unifasciata* is similar to *Dercetina taiwana* and *Dercetina barclayi* sp. n. with the evenly convex pronotum and similar color pattern but differing by antennomere IV much longer than III (1.8 times in *Dercetina unifasciata* in contrast with 1.2 or 1.3 times in *Dercetina taiwana* and *Dercetina barclayi* sp. n.).

#### Redescription.

**Figures 63–68.**
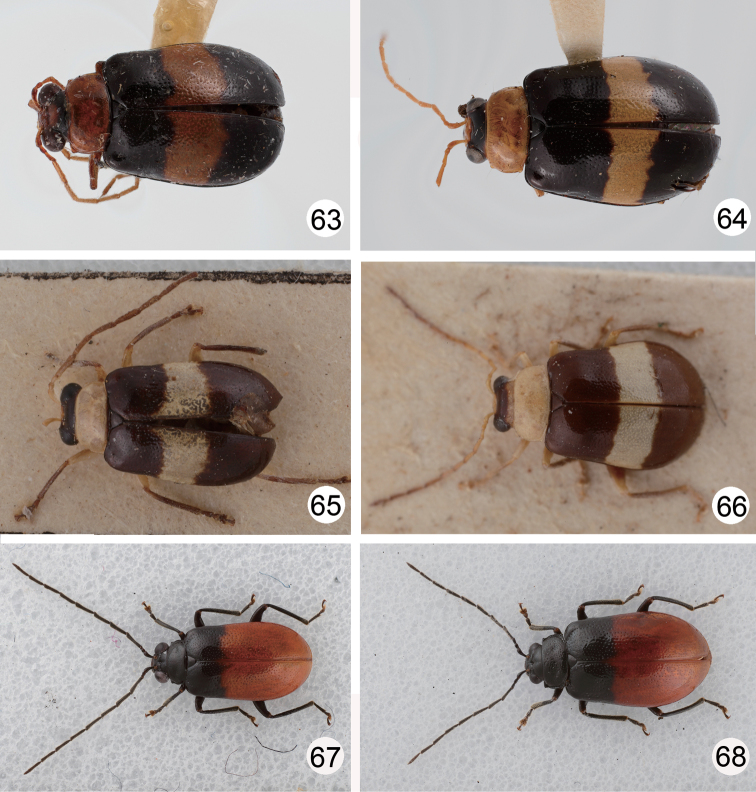
Dorsal habitus of *Dercetina* and *Arthrotus* species. **63** *Dercetina unifasciata*, male **64** *Dercetina unifasciata*, female **65** *Arthrotus flavocincta*, male **66** *Arthrotus flavocincta*, female **67** *Arthrotus nakanei*, male; 68. *Arthrotus nakanei*, female.

Color (Figs 63–64) bluish black, head, prothorax, coxae, and tibiae yellowish brown; elytra with one transverse white stripe at basal 1/3. Head smooth and impunctate. Pronotum transverse, 2.0 times wider than long, evenly convex on disc and lacking fovea or punctured depression, disc with scattered fine punctures; lateral margin almost straight, anterior margin slightly concave, posterior margin slightly rounded. Elytra more or less widened posteriorly, apex convergently rounded, 1.4 times longer than wide, disc with punctures in part arranged in longitudinal rows, epipleurae smooth and impunctate.

**Figures 69–75.**
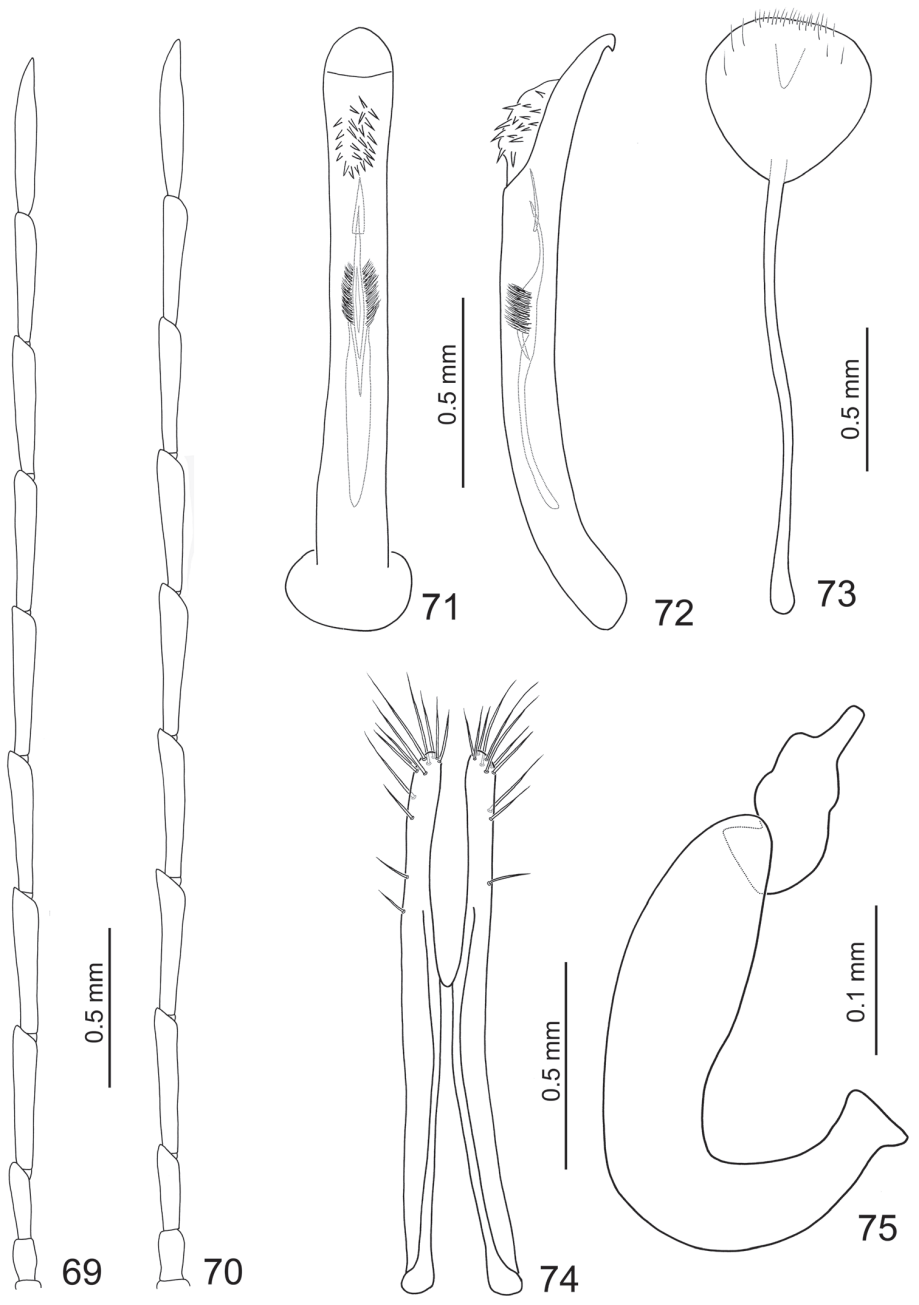
*Dercetina unifasciata*. **69** Antenna, male **70** Antenna, female **71** Aedeagus, dorsal view **71** Aedeagus, lateral view **73** Sternite VIII **74** Gonocoxae **75** Spermatheca.

**Male.** Length 4.7 mm, width 2.5 mm. Ratio of length of antennomeres III to XI about 1.0: 1.8: 1.9: 1.9: 1.9: 1.8: 1.8: 1.7: 2.0; ratio of length to width of antennomeres III to XI about 3.8: 6.0: 6.2: 6.1: 6.3: 5.9: 6.2: 6.3: 7.1 (Fig. 69). Penis (Fig. 71) extremely slender, about 11.0 times longer than wide, parallel-sided, basally and apically widened, apex narrowly rounded; tectum membranous, with scattered long setae; weakly curved in lateral view, apex recurved and pointed (Fig. 72); endophallic sclerites elongate, about 0.6 times as long as penis; apex pointed, ventrally covered with a small apically pointed sclerite; with a cluster of long setae at middle; sinuate in lateral view.

**Female.** Length 4.7–5.8 mm, width 2.7–3.2 mm. Antenna filiform (Fig. 70), similar to male, ratio of length of antennomeres III to XI about 1.0: 1.7: 1.7: 1.7: 1.8: 1.7: 1.6: 1.5: 1.9; ratio of length to width of antennomeres III to XI about 3.9: 6.6: 6.2: 6.3: 6.3: 5.7: 5.7: 5.4: 6.7. Sternite VIII (Fig. 73) weakly sclerotized subapically, apex rounded, with dense short setae along apical margin, spiculum extremely long. Spermathecal receptaculum (Fig. 75) slightly swollen; pump narrow and strongly curved, apex widened and truncate; spermathecal duct short, narrowed in middle, shallowly projecting into receptaculum. Gonocoxae (Fig. 74) very close at middle, about 5.9 times longer than wide, slightly curved inwards near apex, apex rounded, with one or two short setae at apical 1/3, two subapically, eight setae at apex.

#### Distribution.

Cambodia, India, Laos.

### Key to species of the genus *Dercetina* from Taiwan and their similar species

**Table:** 

1	Pronotum with lateral fovea	2
–	Pronotum evenly convex and without lateral fovea	4
2.	Head, prothorax, meso- and metathoracic ventrites yellowish bown	*Dercetina azumai* Gressitt & Kimoto
–	Head, prothorax, meso- and metathoracic ventrites metallic green or purple	3
3	Metallic green; pronotum with prominent punctures	*Dercetina shirozui* Kimoto
–	Metallic purple; pronotum with fine punctures	*Dercetina itoi* Kimoto
4	Antennomere IV much longer than III (1.8 times)	*Dercetina unifasciata* (Allard)
–	Antennomere IV a little longer than III (1.2–1.3 times)	5
5	Elytra oblong (1.6 times longer than wide); elytra yellowish brown with black bands along suture and lateral margins in most individuals	*Dercetina chinensis* (Weise)
–	Elytra oval (1.4 times longer than wide); elytra bluish black with white transverse band at middle in most individuals	6
6	Antenna yellowish brown; endophallic sclerites longer	*Dercetina barclayi* sp. n.
–	Antenna blackish brown, endophallic sclerites shorter	*Dercetina taiwana* (Chûjô)

### Species excluded from the genus *Dercetina*

#### Arthrotus flavocincta

(Hope, 1831) comb. n.

http://species-id.net/wiki/Arthrotus_flavocincta

Galleruca flavocincta Hope, 1831: 29.
Monolepta flavocincta : Weise 1924: 168.
Dercetis flavocincta : Maulik 1936: 355.
Dercetina flavocincta : Kimoto and Takizawa 1972: 222 (Nepal); Kimoto 1989b: 229 (Thailand, Laos, Vietnam).
Antipha flavofasciata Baly, 1879: 456; Maulik 1936: 355. (as synonym of *Dercetina flavocincta*) synonymy confirmed
Dercetes femoralis Weise, 1922: 97; Kimoto 1989: 229 (as synonym of *flavocincta*) synonymy confirmed

##### Type series.

*Galleruca flavocincta*: Holotype ♀ (BMNH), labeled: “Type (circular label with red border) / *Antipha flavocincta* Hope Type (*Galleruca*) / Hardwicke Bequest”.

*Antipha flavofasciata*: Holotype ♀ (BMNH), labeled: “Type (circular label with red border) / Assam / *Antipha flavofasciata* Baly / *Antipha flavofasciata* Baly Cist. Ent. II p. 56 [on the back] / Baly Coll.”.

*Dercetina femoralis*: Holotype ♂ (NHRS): “Tonkin Montes Mauson / April,May 2-3000’ H Frustorfer / femoralis m. / Typus (red label) / NHRS-JLKB 000020341”.

##### Material examined.

**INDIA**: 2♀♀, Assam, 15.VIII.1918 (BMNH); 1♀, Assam, Jorhat, 3721, coll. H. E. Andrewes (BMNH); 1♀, Darjeeling, Bengal, Debrepani, 6000’, 18.IX.1929, leg. J.C.M. Gardner (BMNH); 2♂♂, 1♀, Darjeeling, Bengal, Lopchu, 5000’, 21.IX.1929, leg. J.C.M. Gardner (BMNH); 1♀, Mongpoh, Khasi Hills, 2178, coll. H. E. Andrewes (BMNH); 2♀♀, Sikkim (MUHUB); 2♀♀, Simla (MNHUB); **LAOS**: 1♀, Vientiane Prov., Ban Van Eue, 15.III.1966, leg. Native Collector (BPBM); 1♀, Xiengkhoang Prov., Ban Theuong, 1035 m, 10-17.VIII.1960, leg. R. E. Leech (BPBM); 1♀, Blao (Balao), 500 m, 14–21.X.1960, leg. C. M. Yoshimoto (BPBM); 1♀, Muong Sing, NW of Luang Prabang, 650 m, 6–10.VI.1960, leg. S. Quate & L. Quate (BPBM); **THAILAND**: 1♀, Chiengmai, 3.XII.1962 (KMNH); 2♀♀, Khao Yai Natioal Park, 800–1000m, 18.VIII.1992, leg. D. Furth (USNM); **VIETNAM**: 2♀♀, Dalat, 1500 m, 29.IV.–4.V.1960, leg. C. M. Yoshimoto (BPBM); 1♀, Fyan, 1200 m, 11.VII. –9.VIII.1961, leg. N. R. Spencer (BPBM); 1♀, M’Drak, E of BanMeThuot, 400-600 m, 8–19.1960, leg. C. M. Yoshimoto (BPBM);

##### Diagnosis.

Females of *Arthrotus flavocincta* are similar to *Dercinta unifasciata* with antennomere IV much long than III, but differs by more slender antenomeres IV-VII (4.5–5.7 times longer than wide in *Arthrotus flavocincta* in contrast with 6.0-6.6 times longer than in wide in *Dercetina unifasciata*). Males of *Arthrotus flavocincta* are easily recognized by the similar length of antnnomeres III and IV.

##### Redescription.

Color (Figs 65–66) bluish black, head, prothorax, coxae, and tibiae yellowish brown; elytra with one transverse white stripe in middle; vertex darkened. Head smooth and impunctate. Pronotum transverse, 2.1 times wider than long, evenly convex on disc and lacking fovea or punctured depression, disc with scattered fine punctures; lateral margin straight, anterior margin slightly concave, posterior margin slightly rounded. Elytra parallel-sided, apex convergently rounded, 1.6 times longer than wide, disc with random punctures, epipleurae smooth and impunctate.

**Figures 76–82.**
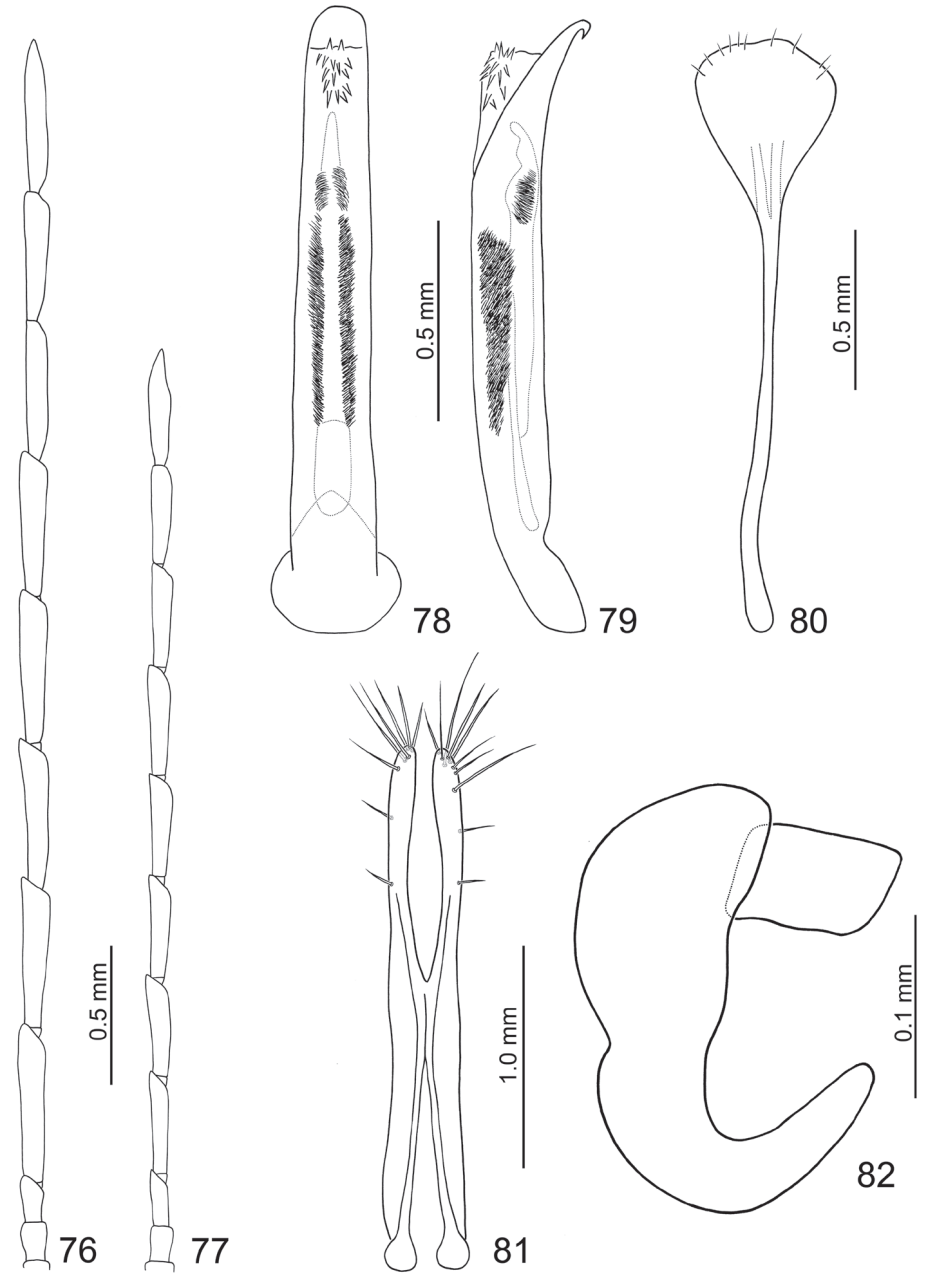
*Arthrotus flavocincta*. **76** Antenna, male **77** Antenna, female **78** Aedeagus, dorsal view **79** Aedeagus, lateral view **80** Sternite VIII **81** Gonocoxae **82** Spermatheca.

**Male.** Length 4.3 mm, width 2.0 mm. Antenna filiform (Fig. 76), antennomere II as long as antennomer III, ratio of length of antennomeres III to XI about 1.0: 3.5: 3.4: 3.3: 3.4: 3.3: 3.0: 2.8: 3.4; ratio of length to width of antennomeres III to XI about 2.1: 5.9: 5.7: 5.7: 5.4: 5.2: 4.5: 4.2: 5.4. Penis (Fig. 78) extremely slender, about 9.4 times longer than wide, parallel-sided, basally widened but apically narrowed; apex narrowly rounded; tectum membranous, with dense long setae; weakly curved in lateral view, apex hooked (Fig. 79); endophallic sclerites elongate, about 0.6 times as long as penis, with a cluster of long setae near apex; in lateral view apex curved; from basal 1/3 to apical 1/3 dorsally covered with a pair of longitudinal areas covered by dense setae.

**Female.** Length 4.4–6.6 mm, width 2.4–3.3 mm. Antenna much shorter than male, comparatively narrower than male (Fig. 77), ratio of length of antennomeres III to XI about 1.0: 1.8: 1.8: 1.8: 1.8: 2.0: 1.8: 1.8: 2.3; ratio of length to width of antennomeres III to XI about 3.1: 5.7: 4.5: 4.6: 4.5: 5.2: 4.9: 5.1: 5.2. Sternite VIII (Fig. 80) weakly sclerotized subapically, apex rounded, setae along lateral and apical margins, spiculum long. Spermathecal receptaculum (Fig. 82) slightly swollen; pump narrow and strongly curved, apex narrowly rounded; spermathecal duct short and wide, shallowly projecting into receptaculum. Gonocoxae (Fig. 81) narrowly connected in middle, extremely elongate, about 6.6 times longer than wide, slightly curved inwards at apical 1/4, with one short setae at apical 1/3, another short setae near apex, six to eight setae at apex.

**Color variation.** Some specimens have blackish brown meso- and metacoxae, femora, and vertex, or yellowish brown abdomen.

##### Distribution.

India, Laos, Nepal, Thailand, Vietnam.

#### Arthrotus nakanei

(Kimoto, 1969) comb. n.

http://species-id.net/wiki/Arthrotus_nakanei

Dercetina nakanei Kimoto, 1969: 65.

##### Type series.

Holotype ♀ (KUEC): “(Taiwan) Sungkan, 2000m – Tsifeng, 2300m Nantou Hsien / 29.vi.1965 T. Nakane / Japan-U.S. Co-op. Sci. Programme (yellow label) / *Dercetina nakanei* Kimoto, n. sp. / HOLOTYPE (red label)”. Paratypes: 1♀ (KMNH): “(Taiwan) Sungkang Nantou Hsien / 19.v.1965 T. Shirôzu / *Dercetina nakanei* Kimoto, n. sp. / PARATYPE (blue label)”; 1♀ (KMNH): “(Taiwan) Meifeng Nantou Hsien / 18.v.1965 B.C. Chang / *Dercetina nakanei* Kimoto, n. sp. / PARATYPE (blue label)”.

##### Material examined.

**TAIWAN**: 1♀, Hsinchu, Kuanwu, 6.IV.2010, leg. L.-H. Sun (TARI); 5♂♂, 8♀♀, same locality, 30.IV.–1.V.2010, leg. M.-H. Tsou (TARI); 1♂, 1♀, same locality, 30.IV.2010, leg. C.-F. Lee (TARI); 1♀, same locality, 7.VI.2010, leg. L.-H. Sun (TARI); 5♂♂, Nantou, Chingching, 4.IV.2010, leg. Y.-T. Wang (TARI); 6♂♂, 7♀♀, Nantou, Meifeng, 15.IX.2009, leg. H. Lee (TARI); 1♂, same locality, 20.IV.2011, leg. C.-F. Lee (TARI); 2♀♀, Nantou, Sungkang, 4.IV.2010, leg. Y.-T. Wang (TARI); 1♂, 1♀, Nantou, Tsuifeng, 5.IV.2010, leg. Y.-T. Wang (TARI); 1♂, Taichung, Anmashan (= Tashueshan), 15.X.2009, leg. J.-C. Chen (TARI); 1♀, same locality 19.X.2011, leg. C.-F. Lee (TARI); 1♀, same locality, 24.IV.2012, leg. C.-F. Lee; 1♂, Taichung, Henglingshan, 5.VI.2012, J.-C. Chen (TARI);

##### Diagnosis.

*Arthrotus nakanei* is easily recognized by its color pattern.

##### Redescription.

Color (Figs 67–68) black, abdomen and apical 2/3 of elytra reddish brown. Head smooth and impunctate. Pronotum transverse, 1.9–2.0 times wider than long, disc with scattered prominent punctures, and a pair of round fovea at sides; lateral and posterior margins slightly rounded, anterior margin slightly concave. Elytra more or less widened posteriorly, apex convergently rounded, 1.6–1.7 times longer than wide, disc with punctures in part arranged in longitudinal rows, epipleurae smooth and impunctate.

**Figures 83–89.**
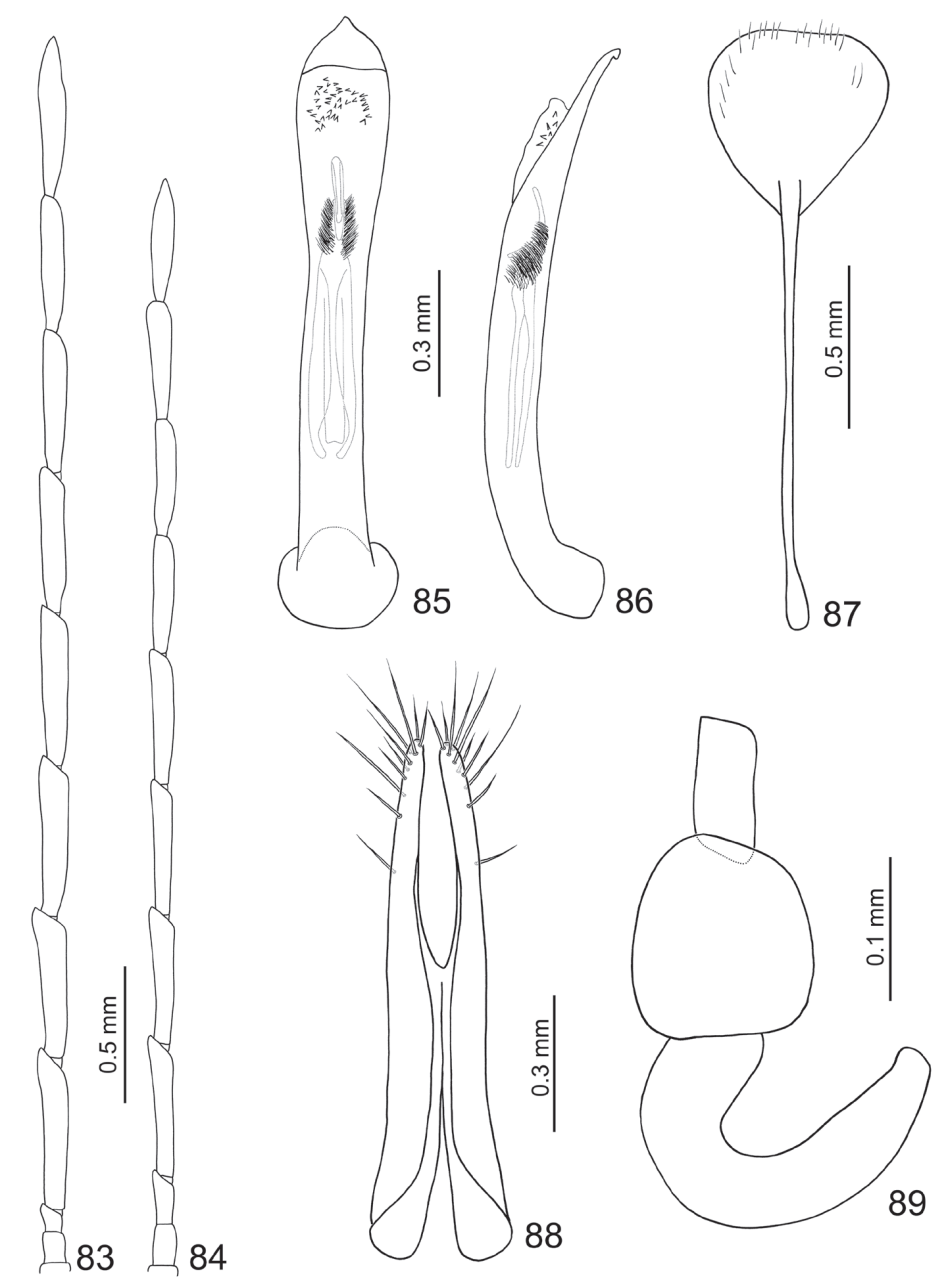
*Arthrotus nakanei*. **83** Antenna, male **84** Antenna, female **85** Aedeagus, dorsal view **86** Aedeagus, lateral view **87** Sternite VIII **88** Gonocoxae **89** Spermatheca.

**Male.** Length 5.2–5.7 mm, width 2.4–2.7 mm. Antenna filiform (Fig. 83), antennomere II a little smaller than III, ratio of length of antennomeres III to XI about 1.0: 5.7: 5.3: 5.6: 5.6: 5.1: 5.1: 5.1: 5.6; ratio of length to width of antennomeres III to XI about 1.2: 5.1: 4.8: 5.3: 5.6: 5.4: 5.7: 5.7: 5.6. Penis (Fig. 85) extremely slender, about 8.9 times longer than wide, lateral margin medially narrowed, apex narrowly rounded, with a short process at top; tectum membranous, with scattered short setae; weakly curved in lateral view, apex recurved and hooklike (Fig. 86); endophallic sclerites elongate, about 0.5 times as long as penis; apically narrowed, apex ventrally covered with a small curved sclerite; with a cluster of long setae from middle to apex; straight in lateral view.

**Female.** Length 6.0–6.2 mm, width 2.9–3.2 mm. Antenna filiform (Fig. 84), shorter than male, ratio of length of antennomeres III to XI about 1.0: 2.5: 2.4: 2.4: 2.4: 2.1: 2.1: 2.1: 2.2; ratio of length to width of antennomeres III to XI about 2.3: 5.9: 5.7: 5.7: 5.7: 5.3: 5.2: 5.2: 5.5. Sternite VIII (Fig. 87) weakly sclerotized subapically, apex truncate, with dense long setae along apical margin, spiculum extremely long. Spermathecal receptaculum (Fig. 89) strongly swollen; pump narrow and strongly curved, apically narrowed; spermathecal duct short, shallowly projecting into receptaculum. Gonocoxae (Fig. 88) very close at middle, about 5.0 times longer than wide, slightly curved inwards near apex, apex rounded, with one or two short setae at apical 1/3, two subapically, nine setae at apex.

##### Distribution.

Taiwan.

## Discussion

Endophallic sclerites is useful for species identities but not for generic diagnosis for *Dercetina* and *Arthrotus*. The extreme narrow connection between gonocoxae seems to characterize the genus *Arthrotus* based on the studies on two member of the genus, although this character should be evaluated after more species are studied.

## Supplementary Material

XML Treatment for Dercetina azumai

XML Treatment for Dercetina barclayi

XML Treatment for Dercetina chinensis

XML Treatment for Dercetina itoi

XML Treatment for Dercetina shirozui

XML Treatment for Dercetina taiwana

XML Treatment for Dercetina unifasciata

XML Treatment for Arthrotus flavocincta

XML Treatment for Arthrotus nakanei
